# Gene Co-Expression Analysis Reveals Transcriptome Divergence between Wild and Cultivated Sugarcane under Drought Stress

**DOI:** 10.3390/ijms23010569

**Published:** 2022-01-05

**Authors:** Peiting Li, Pingping Lin, Zhenli Zhao, Zihong Li, Yanming Liu, Chaohua Huang, Guoqiang Huang, Liangnian Xu, Zuhu Deng, Yu Zhang, Xinwang Zhao

**Affiliations:** 1 National Engineering Research Center for Sugarcane, Key Laboratory of Sugarcane Biology and Genetic Breeding Ministry of Agriculture, Fujian Agriculture and Forestry University, Fuzhou 350002, China; lipeiting147258@163.com (P.L.); opingping2021@163.com (P.L.); zzl135896@163.com (Z.Z.); lizihong00@163.com (Z.L.); Lym981126@163.com (Y.L.); ganzhe403@163.com (C.H.); hgq94@163.com (G.H.); xuliangnian@163.com (L.X.); dengzuhu@163.com (Z.D.); 2 Fujian Provincial Key Laboratory of Plant Functional Biology, College of Life Sciences, Fujian Agriculture and Forestry University, Fuzhou 350002, China; 3 Guangxi Key Laboratory for Sugarcane Biology, Guangxi University, Nanning 530005, China

**Keywords:** sugarcane, drought resistant, transcriptome, WGCNA, transcription factor

## Abstract

Drought is the main abiotic stress that constrains sugarcane growth and production. To understand the molecular mechanisms that govern drought stress, we performed a comprehensive comparative analysis of physiological changes and transcriptome dynamics related to drought stress of highly drought-resistant (ROC22, cultivated genotype) and weakly drought-resistant (Badila, wild genotype) sugarcane, in a time-course experiment (0 h, 4 h, 8 h, 16 h and 32 h). Physiological examination reviewed that ROC22, which shows superior drought tolerance relative to Badila, has high performance photosynthesis and better anti-oxidation defenses under drought conditions. The time series dataset enabled the identification of important hubs and connections of gene expression networks. We identified 36,956 differentially expressed genes (DEGs) in response to drought stress. Of these, 15,871 DEGs were shared by the two genotypes, and 16,662 and 4423 DEGs were unique to ROC22 and Badila, respectively. Abscisic acid (ABA)-activated signaling pathway, response to water deprivation, response to salt stress and photosynthesis-related processes showed significant enrichment in the two genotypes under drought stress. At 4 h of drought stress, ROC22 had earlier stress signal transduction and specific up-regulation of the processes response to ABA, L-proline biosynthesis and MAPK signaling pathway–plant than Badila. WGCNA analysis used to compile a gene regulatory network for ROC22 and Badila leaves exposed to drought stress revealed important candidate genes, including several classical transcription factors: *NAC87*, *JAMYB*, *bHLH84*, *NAC21/22*, *HOX24* and *MYB102*, which are related to some antioxidants and trehalose, and other genes. These results provide new insights and resources for future research and cultivation of drought-tolerant sugarcane varieties.

## 1. Introduction

Drought is one of the most important environmental factors that threatens agricultural production worldwide. Limited water resources have exacerbated the impact of arid climate conditions on crop production [1]. Sugarcane (*Saccharum* spp.) is an important sugar and energy crop that has strict water requirements for cultivation [2].

Drought stress can damage plant enzyme activity and reduce cell turgor and energy supply, as well as inhibit mitosis and normal cell metabolism. All of these effects can hinder normal growth of crops [3]. Osmotic regulation and antioxidant defense are important physiological events in the resistance of plants to drought stress damage. Plants can accumulate various organic and inorganic substances (e.g., mannitol, proline, glycine, betaine, trehalose, fructan, inositol and inorganic ions) in cells that are important to maintain physiological activities during long-term drought [4]. The antioxidant defense system involves the enzyme components superoxide dismutase, catalase (CAT), peroxidase (POD), ascorbic acid peroxidase, glutathione peroxidase (GPX) and glutathione reductase (GR) as well as non-enzymatic components such as cysteine, reduced glutathione and ascorbic acid [5].

Drought resistance is a complex trait that is controlled by many mechanisms. ABA is a key hormone of plants, regulating the physiological and molecular reactions that respond to drought stress, such as stomatal closure, gene expression, osmotic protectant, and stress protein accumulation. During the stress response, ABA must also mediate crosstalk with other signal pathways to perform its function. For example, ABA activates brassinosteroid-intrinsic 2 (BIN2), a negative regulator of brassinosteroid signaling, by inhibiting ABA INSENSITIVE1 (ABI1) and ABI2-mediated BIN2 dephosphorylation. BIN2 phosphorylates SnRK2s and activates downstream pathways through the central role of SnRK2s in the ABA signaling pathway [6]. ABA activates SnRK2s to phosphorylate ABA responsive kinase substrate 1 (ASK1) to inhibit its transcriptional activity. Then, multiple reactions lead to the inhibition of K^+^ influx-mediated stomatal opening that promotes stomatal closure in response to ABA [7]. The mitogen activated protein kinase (MAP) cascade also plays an important role in plant drought stress response. This signaling pathway transmits pressure signals from receptors to specific effectors to regulate gene expression, cell activity and protein function in various developmental and environmental adaptation processes [8].

Transcription factors are another group of indispensable regulatory proteins in plants. They regulate gene expression in response to drought stress at the transcriptional level. The bZIP transcription factor ABRE-binding proteins (AREBs)/ABFs(ABFs) can bind to the ABA response element (ABRE, PyACGTGGC) and activate downstream genes. Indeed, the ABRE element is an enriched motif in promoters of drought response genes [9]. *OsMYB2* expression is induced by a variety of stresses and increases the tolerance of transgenic plants to salt, cold and dehydration stresses by regulating accumulation of H_2_O_2_ and malondialdehyde as well as the expression of genes involved in proline synthesis [10]. NAC transcription factors are involved in almost all aspects of plant activities throughout the plant life cycle. Overexpression of *OsNAC10* significantly expands the root diameter of transgenic rice and therefore enhances drought tolerance under normal and drought stress conditions during the reproductive stage [11]. The regulatory networks of the related transcription factors were exhibited well by weighted gene co-expression network analysis (WGCNA) [12].

Sugarcane has strict water requirements and thus is sensitive to drought stress. In this study, we performed a comprehensive comparative analysis of physiological changes and transcriptomes of the highly drought-resistant genotype ROC22 and weakly drought-resistant genotype Badila sugarcane exposed to drought stress in a time-course experiment (0 h, 4 h, 8 h, 16 h and 32 h). We also examined the adaptive characteristics of sugarcane with increasing drought stress time to reveal potential adaptive mechanisms by transcriptome-based WGCNA analysis. Our findings expand our understanding of sugarcane adaptation to drought stress and provide valuable information for improving drought tolerance of sugarcane.

## 2. Results

### 2.1. Morphological and Phenotypic Changes of Sugarcane Seedlings Exposed to Drought

Based on genetic relationships and inferred differences in drought resistance, we selected two sugarcane genotypes, *Saccharum officinarum* L. (Badila) and a cultivated variety, ROC22. According to previous studies, the ROC22 sugarcane cultivar has strong drought resistance, whereas Badila exhibits weak drought resistance [13].

**Figure 1:**
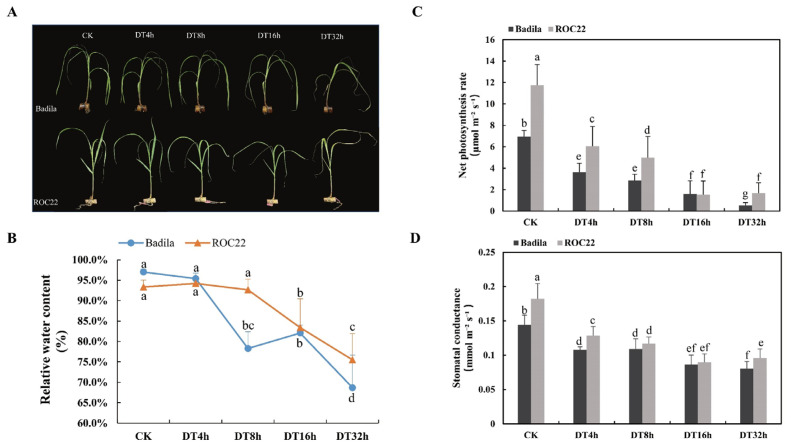
Effect of 20% PEG6000 stress on sugarcane. (**A**) The 20% PEG6000 simulated the phenotypic changes of ROC22 and Badila under drought stress. (**B**) Broken line diagram the relative water content RWC (%) of leaves. (**C**) Broken line diagram net photosynthetic rate. (**D**) Broken line diagram of stomatal conductance of different samples. Different lowercase letters indicate that there is a significant difference between the mean values (one-way ANOVA with Ducan’s multiple range test, *p* ≤ 0.05). CK, control group, DT4h, drought treatment for 4 h, DT8h, drought treatment for 8 h, DT16h, drought treatment for 16 h and DT32h, drought treatment for 32 h, *n* = 15.

To obtain transcriptome data for sugarcane leaves exposed to drought stress and for varying exposure times, ROC22 and Badila seedlings were treated with 20% PEG6000 for 32 h. After 4 h, a small part of the leaf tips of both sugarcane types had curled and yellowed due to water loss. However, compared with the untreated control, there were no obvious differences across the whole plant. After 8 h, the curl and shrinkage of the blade tip increased plants. After 16 h, the leaves of both Badila and ROC22 began to wither and shrink in large segments away from the leaf tip. After 32 h of drought stress treatment, both Badila and ROC22 showed leaf chlorosis, drooping, shrinkage and wilting. Meanwhile, the stem of ROC22 remained upright similar to that seen for the 8- and 16-h treatments, while the stem of Badila obviously began to soften, lose support and bend (Figure 1A).

We measured the relative water content (RWC) of leaves from the two plant types at each time point. ROC22 had significantly lower rates of water loss than Badila. The RWC of the two genotypes did not changes significantly during the first 4 h of drought stress, but the RWC of Badila decreased significantly after 8 h, while ROC22 did not change significantly during this period. At 16 h, the ROC22 RWC began to decrease significantly and at 32 h, the RWC of both Badila and ROC22 decreased significantly, although the RWC for ROC22 was significantly higher than that for Badila (Figure 1B).

We measured the net photosynthetic rate and stomatal conductance of Badila and ROC22 with different times of drought exposure. As the exposure time increased, the net photosynthetic rate and stomatal conductance of Badila and ROC22 decreased gradually, but values for both factors were generally higher for ROC22 compared to Badila. Together, the results of this comprehensive analysis showed that the drought tolerance of ROC22 was stronger than that of Badila, particularly in the later stages of drought stress (Figure 1C,D).

### 2.2. Overview of Transcriptome Divergence between Wild and Cultivated Sugarcane

To investigate transcriptional processes that diverge between ROC22 and Badila under drought stress, we generated RNA-seq data from the two genotypes at 5 time points (0 h, 4 h, 8 h, 16 h and 32 h) after PEG6000 (20% *w*/*v*) treatment with three biological repeats. Thirty pair-end libraries with a total of 299.01 Gb clean data were obtained. The average clean data of each library reached 7.91 Gb, and the percentage of Q30 bases was 93.15% to 94.69%. Sequence alignment with sugarcane reference genome showed that the alignment efficiency ranged from 71.03% to 86.50% (Table S2). A total of 35,082 genes were obtained when the FPKM was greater than 1. For subsequent analysis, these samples are designated as “B/Rtreatment behavior”. For example, RCK represents ROC22 control treatment, RDT4 represents ROC22 PEG6000 stress treatment for 4 h, and samples at other treatment time points also use similar abbreviations.

**Figure 2:**
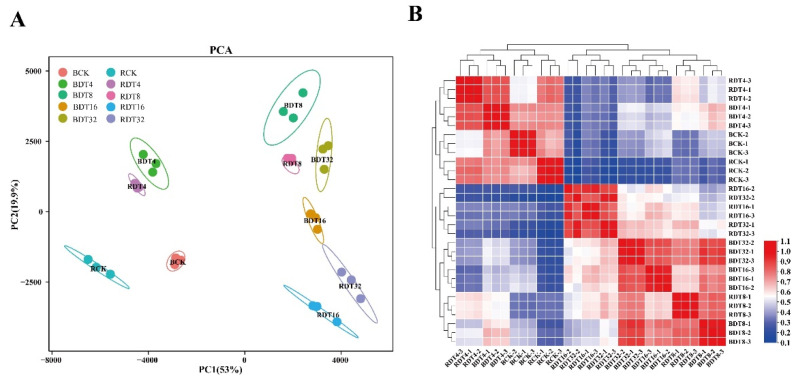
Transcriptome differences between two genotypes of sugarcane (ROC22 and Badila). (**A**) PCA diagram of sample expression at different time points of ROC22 and Badila drought stress. (**B**) In the cluster heatmap of the correlation of the expression quantity of all samples, red indicates high correlation and blue indicates low correlation.

Principal component analysis (PCA) and clustering heatmap analysis of the transcriptomic data revealed a high similarity among the three biological replicates within each treatment (Figure 2). Among treatments, a clear separation of ROC22 control from Badila control plants was observed, while a closer distance between RDT4 and BDT4 was observed (Figure 2A). Interestingly, over the 8–32 h time course, the transcriptome of Badila and ROC22 became progressively divergent under drought stress (Figure 2A). Sample clustering heatmap analysis also exhibited a similar time point clusters (Figure 2B). The results showed that the gene expression patterns of Badila and ROC22 under short-term (4 h) drought stress and control condition is similar, but there are great transcriptome differences between the two genotypes under long-term (8–32 h) drought stress.

### 2.3. Temporal Variation of Transcriptome of Different Drought Resistant Materials under Drought Stress

A comparison of untreated ROC22 samples (RCK) with untreated Badila samples (BCK) highlighted 13,886 differentially expressed genes (DEGs), including 8621 up-regulated DEGs and 5265 down-regulated DEGs. GO enrichment analysis showed that genes in five processes “defense response”, “cell cycle”, “cellular carbohydrate biosynthetic process”, “cell wall organization” and “photosynthesis” were up-regulated. This result suggests that strong photosynthetic capacity and active cell wall composition activity may be present in drought-tolerant genotypes. “Transposition, DNA-mediated”, “telomere maintenance”, “DNA recombination”, “DNA integration” and “gene silencing by RNA” were significantly down-regulated and enriched. This finding shows that gene transcription and translation processes occurred continuously in Badila under drought stress (Table S3).

**Figure 3:**
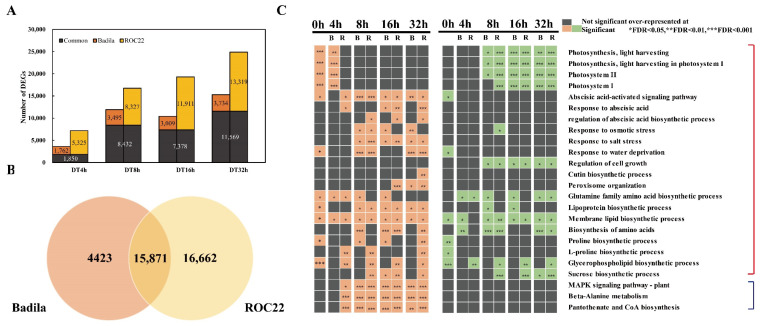
Temporal changes of response transcriptome in sugarcane leaves. (**A**) The number of DEGs of Badila and ROC22 after drought stress is compared between treated samples and untreated samples. 0 h (untreated samples), DT4h (drought treatment for 4 h), DT8h (drought treatment for 8 h), DT16h (drought treatment for 16 h), DT32h (drought treatment for 32 h). (**B**) Venn diagram of DEGs of Badila and ROC22 under drought stress. (**C**) The differentially expressed genes KEGG and GO of the two materials are enriched. B (Badila), R (ROC22); 0 h indicates the DEGs between ROC22 and Badila without treatment, 4 h, 8 h, 16 h, 32 h indicates the DEGs drought treatment for 4 h, 8 h, 16 h and 32 h; orange on the left is the annotation of up-regulated genes, green on the right is the annotation of down-regulated genes; the red line indicates the GO terms, the blue line indicates the KEGG pathway.

The DEGs under drought treatment were identified by comparing each treatment time point with 0 h (FDR ≤ 0.05 and |log2FC| ≥ 2). For Badila, 3612, 11,927, 10,387 and 15,303 DEGs were identified at BDT4, BDT8, BDT16 and BDT32, respectively. For ROC22, 7175, 16,759, 19,289 and 24,888 DEGs were identified at RDT4, RDT8, RDT16 and RDT32, respectively. The number of DEGs increased with the drought treatment time for both genotypes, except for DT16h in Badila. More DEGs were obtained for ROC22 than for Badila at each treatment time point (Figure 3A). A total of 15,871 DEGs associated with response to drought stress overlapped in both genotypes, with 4423 and 16,662 DEGs specially expressed in Badila and ROC22, respectively (Figure 3B).

To gain insight into the biological processes that were commonly or uniquely overrepresented between ROC22 and Badila in response to drought stress, we analyzed GO and KEGG enrichment of DEGs. The up-regulated common biological processes included “response to abscisic acid”, “abscisic acid-activated signaling pathway”, “response to water deprivation”, “response to osmotic stress” and “peroxisome organization” terms (Figure 3C). Meanwhile, photosynthesis-related and “regulation of cell growth” processes were down-regulated in both ROC22 and Badila under drought stress (Figure 3C). Membrane lipid, lipoprotein and glutamine family amino acid biosynthetic processes were very active, as evidenced by enrichment of GO terms in both up- and down-regulated DEGs, as well as in the two sugarcane varieties. KEGG enrichment analysis showed that “MAPK signaling pathway-plant”, “beta-Alanine metabolism” and “pantothenate and CoA biosynthesis” pathways were also up-regulated and enriched in the two genotypes (Figure 3C).

After drought stress, the up-regulated DEGs in ROC22 were specifically enriched in “regulation of abscisic acid biosynthetic process”, “cutin biosynthetic process” and “L-proline biosynthetic process”. “Glycerophospholipid biosynthetic process” is active in ROC22 and was specifically enriched in up-regulated and down-regulated genes. The up-regulated DEGs of Badila after 16 and 32 h of stress were specifically enriched in “response to osmotic stress”, which indicates that the cell osmotic pressure of Badila changes sharply in the late stage of drought, and more genes are needed to respond to osmotic stress (Figure 3C).

Several results attracted our attention. Compared with Badila, some changes in some processes occurred earlier and were more lasting in ROC22 after stress. For example, “abscisic acid activated signaling pathway” was significantly up-regulated and enriched 4 h after ROC22 stress, while in Badila similar changes began 8 h after stress (Figure 3C). The up-regulated DEGs of RDT4 and RDT32 were significantly enriched in the “responses to abscisic acid” process. The up-regulated DEGs of RDT8 and RDT32 were significantly enriched in the “sucrose biological process”, and the up-regulated DEGs of RDT16 were significantly enriched in “peroxisome organization”, which was not seen in Badila (Figure 3C). ROC22 also showed specific up-regulation and enrichment in “MAPK signal pathway–plant” and “pantothenate and CoA biosynthesis” pathways after 4 h of stress, which was earlier than Badila. Expression ABA, MAPK and peroxisome-related genes were up-regulated faster and more persistently in ROC22, which may contribute to the drought resistance of this genotype. This series of genotype-specific enrichment biological processes/pathways may be closely related to the different drought resistance of sugarcane.

### 2.4. Co-Expression Network Revealed Modules with Different Expression Patterns under Drought Treatment

**Figure 4:**
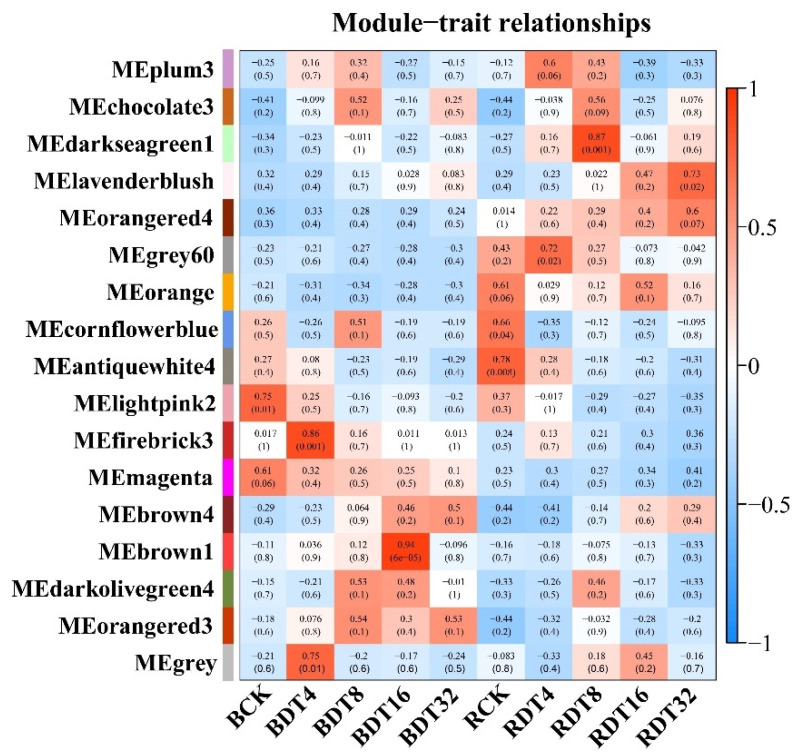
WGCNA analysis of differentially expressed genes in sugarcane. The module sample correlation and corresponding *p* values are shown in parentheses. The panel on the left shows 17 modules. The color code on the right shows the module feature correlation −1 (blue) to 1 (red).

To investigate relationships among drought-stress-response genes, we constructed a weighted co-expression network analysis of all DEGs under drought treatment in chronological order. We obtained 17 modules, which were defined by different color codes. The modules with correlation coefficient ≥ 0.65 and *p* value ≤ 0.05 are defined as sample-specific modules. The relevant combinations of modules and samples are: RCK (antiquewhite4 and cornflowerblue), RDT4 (grey60), RDT8 (darkseagreen1), RDT32 (lavenderblush), BCK (lightpink2), BDT4 (firebrick3 and grey) and BDT16 (brown1). The patterns of some modules may be related to adaptation processes that are activated in response to drought stress (Figure 4).

Modules colored antiquewhite4 (2809), cornflowerblue (36) and lightpink2 (1390) had the highest correlation with RCK and BCK samples. The genes of modules with high similarity to the sample have dominant expression in that sample. The overall expression of the genes of the three modules decreased with increasing stress time (Table 1). GO enrichment analysis showed that the genes in the three modules were significantly enriched in cell membrane lipid related processes (“phospholipid biosynthetic process”, “membrane lipid biosynthetic process” and “glycerophospholipid biosynthetic process”), indicating that expression of the membrane lipid synthesis genes of sugarcane was inhibited as stress treatment progressed. The genes in RCK sample-specific modules (antiquewhite4 and cornflowerblue) were specifically enriched in the “hydrogen peroxide biosynthetic process”. Hydrogen peroxide causes plants to suffer oxidative stress and the inhibition of expression of related synthetic genes may be due to the superior performance of the antioxidant capacity of ROC22. BCK sample-specific module (lightpink2) genes were specifically enriched in some biological processes involving galactolipid (“galactolipid biosynthetic process” and “galactolipid metabolic process”) (Table 1). Galactolipids are a class of membrane lipids in plant cells, and changes in their synthetic and metabolic genes may predict changes in the membrane structure of Badila in the stress process. Some cell growth- and development-related processes were specifically enriched (“regulation of cell growth” and “cellular developmental process”) in Badila. Appropriate regulation of tissue cell growth and development may be a strategy used by Badila to survive under drought stress (Table 1). In addition, it is obvious that more genes in ROC22 were significantly enriched in pathways related to photosynthesis (“photosynthesis, photosynthesis–antenna proteins” and “porphyrin and chlorophyll metabolism”). This finding showed that the photosynthesis-related genes of the two genotypes were inhibited to varying degrees during drought stress (Table 1).

Grey60 (1110), firebrick 3 (67) and grey (52) modules had the highest correlation with RDT4 and BDT4 samples, respectively. The expression of genes in these three modules first increased and then decreased, with the highest expression seen at DT4h (Table 1). Genes of the RDT4 sample-specific module were specifically enriched in cellulose biosynthetic processes (“cellulose microfibril organization”, “plant-type cell wall cellulose biosynthetic process”, “regulation of cellulose biosynthetic process”), which indicates that expression of cellulose biosynthetic genes is inhibited in the late stress stage, suggesting that inhibition of genes related to cellulose synthesis may be a drought tolerance regulatory mechanism in ROC22. The gene specificity in firebrick 3 and the grey module is significantly enriched in “glucose-6-phosphate dehydrogenase activity” (Table 1). The gene specificity of the grey60 module was significantly enriched in the “phenylpropanoid biosynthesis” pathway. Secondary metabolites produced during phenylpropane metabolism are critical for plant drought tolerance (Table 1).

Darkseagreen1 (157) had the highest correlation with RDT8 samples, and the module gene was preferentially expressed in RDT8 before a downward trend occurred at 8–16 h that was followed by a slight increase at 16–32 h (Table 1). Three GO terms encompassing “regulation of brassinosteroid mediated signaling pathway”, “negative regulation of brassinosteroid mediated signaling pathway” and “steroid biosynthetic process” related genes were significantly enriched, indicating that the signal transduction associated with brassinolide is complex throughout the stress process (Table 1).

Brown1 (49) is the module that had the highest similarity with the BDT16 sample, and both genotypes were preferentially expressed with 16 h stress treatment (Table 1). GO enrichment analysis showed that the module gene was significantly enriched in “tRNA metabolic process”, “intra-Golgi vesicle-mediated transport” and “cis-Golgi network membrane”, indicating that an active gene translation process may occur at this time point in response to drought stress.

The largest module is the lavenderblush module (4231), which contains 4231 DEGs and had the highest correlation with RDT32 (Table 1). The expression of DEGs increased in the two genotypes with increasing treatment time, and the gene expression in ROC22 was higher than that for Badila after 4 h of drought stress. The “peroxisome” process was significantly enriched, suggesting that the POD enzyme gene was gradually up-regulated during stress in response to stress responses. In addition, DEGs were significantly enriched in “glucose transport” and “monosaccharide transport” processes, indicating that ROC22 redistributes and utilizes sugars to survive during stress. The DEGs in this module are also involved in fatty acid β-oxidation, lipid oxidation and fatty acid degradation, indicating that oxidative damage to membrane lipids in sugarcane leaves may occur throughout the drought stress process (Table 1).

### 2.5. Complex Signal Transduction Processes Caused by Drought Stress

Plant hormone signal transduction is the main signal transduction process in plants under drought stress. GO enrichment analyses found that the DEGs were significantly enriched in a variety of hormone processes (Figure 3C and Table 1). To further analyze the regulatory effects of early and late signal transduction during drought stress, we studied the related genes.

**Figure 5:**
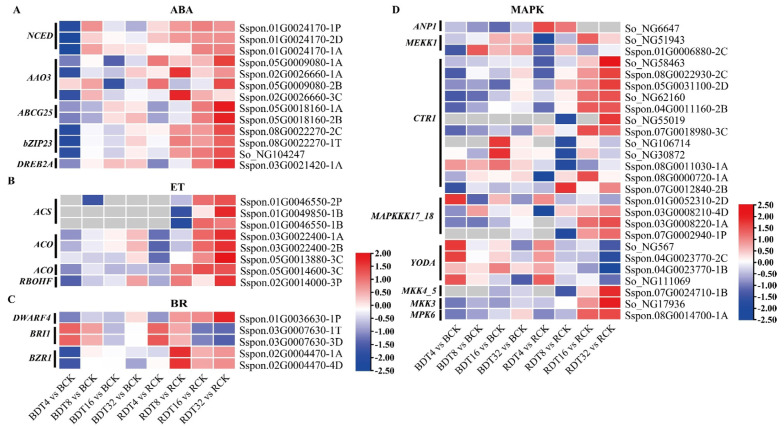
Complex signal transduction pathways under drought stress. (**A**–**C**) The expression patterns of genes related to plant hormone biosynthesis and signal transduction under drought stress. (**D**) The expression patterns of DEGs involved in MAPK signal cascade were mapped by log_2_(FC), and row standardized. Red is up-regulated expression and blue is down-regulated expression.

We identified the expression of key enzyme genes of ABA biosynthesis, 9-cis-epoxy carotenoid dioxygenase gene 1 (*NCED1*) and ABA aldehyde oxidase gene 3 (*AAO3*) in the two genotypes under drought stress. In the expression pattern diagram comparing ROC22 with Badila, the *NCED1* gene in ROC22 was up-regulated after stress before that in Badila, and expression of the *AAO3* gene in ROC22 was up-regulated more than 8 h after stress (Figure 5A). In conclusion, after drought stress, ROC22 was more likely to produce ABA earlier and more continuously than Badila. We found that *ABCG25*, which encodes the ABC transporter G family member 25 protein, was up-regulated in ROC22 under drought stress, but not in Badila (Figure 5A). Moreover, the ABA response element binding factor *bZIP23* was more highly expressed and up-regulated in ROC22 than Badila after stress (Figure 5A). We found that drought stress induced up-regulation of DREB2A [14] in ROC22, but its expression did not change in Badila (Figure 5A).

We found that ACS and ACO, the key enzymes of ethylene synthesis in sugarcane, were up-regulated by drought stress, especially in ROC22 compared with Badila (Figure 5B). This result suggests that drought may induce ethylene production in sugarcane. *ETR2*, a gene encoding ethylene receptor protein 2, was also up-regulated in ROC22, but not in Badila in response to drought stress (Figure 5B). The *RBOHF* gene encoding the nicotinamide adenine dinucleotide phosphate hydrogen (NADPH) oxidase is regulating ethylene induced stomatal closure, the *RBOHF* was particularly up-regulated in ROC22, the expression and up-regulation was multiple-fold higher than that in Badila (Figure 5B).

We found that the gene *DWARF4*, encoding the rate limiting enzyme Cytochrome P450 90B2 of brassinosteroid (BR) biosynthesis, was up-regulated in sugarcane after drought stress. It was found that both the expression amount and the up-regulation multiple of this gene in ROC22 were better than that of Badila (Figure 5C). We also found that the BR receptor kinase *BRI1* gene in ROC22 was inhibited after 16 and 32 h of stress treatment. Moreover, BRASSINAZOLE-RESISTANT1 (*BZR1*) homologous gene, an important transcription factor in brassinosteroid signal transduction, was up-regulated after 8 h of drought stress in ROC22 (Figure 5C).

The mitogen activated protein kinase (MAPK) cascade is the main component downstream of receptors or sensors. MAPK can transform extracellular stimuli such as drought, heat and cold, and ROS into intracellular reactions. Our previous analysis found that up-regulated DEGs were significantly enriched in the MAPK pathway within 0 to 32 h of drought stress. Analysis of genes related to the MAPK cascade in sugarcane drought response showed that the *ANP1* (MAPKKKs) gene in ROC22 was induced in the early stage of stress, whereas three *MEKK1* (MAPKKKs) genes were induced in the early stage of Badila stress (Figure 5D). *CTR1* (MAPKKKs) genes associated with multiple ethylene signal transduction pathways were identified, and were mainly induced and expressed in the late stage of ROC22 stress (Figure 5D). *YODA* (MAPKKKs) is involved in regulating stomatal opening and closing and its expression was induced in the early stage of stress and inhibited at 8 h in ROC22, while Badila was higher expression (Figure 5D). *MAP3K17_18* were induced in the late stage of ROC22 stress, and several were induced in the early stage of Badila stress (Figure 5D). *MKK4_5*, *MKK3* and *MPK6* were induced in the late stage of ROC22 stress. Together, these results show that the MAPK cascade is an important signal transduction process in drought response of sugarcane (Figure 5D).

### 2.6. Enhancement of Antioxidant Capacity of Sugarcane under Drought Stress

**Figure 6:**
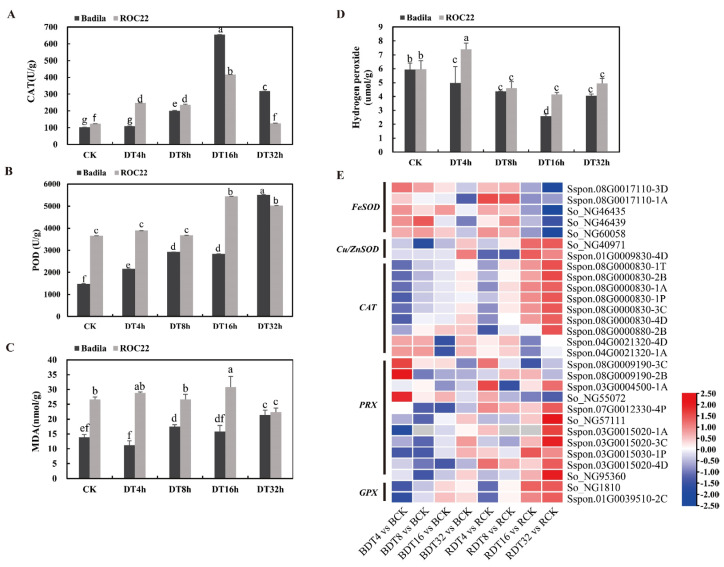
Antioxidant defense system of sugarcane under drought stress. (**A**) CAT enzyme activities of Badila and ROC22 under different drought stress treatment times. (**B**) POD enzyme activity. (**C**) MDA content. (**D**) Hydrogen peroxide content. Different lowercase letters indicate significant differences, *p* ≤ 0.05. (**E**) The expression patterns of genes related to antioxidant enzyme in sugarcane under drought stress were mapped by log_2_(FC), and the data were row standardized. Red was the up-regulated expression and blue was the down-regulated expression.

Activating the antioxidant defense system is important for plants to resist damage due to drought stress. We measured the POD and CAT enzyme activities of the two genotypes under different treatment times, and determined the content of malondialdehyde (MDA) and hydrogen peroxide (H_2_O_2_) in the leaves. Levels of CAT and POD activity and MDA content were higher in untreated ROC22 than untreated Badila. Moreover, the antioxidant activity was higher, and the drought tolerance of ROC22 was improved relative to Badila. With increasing drought time, the MDA content and POD and CAT enzyme activities increased continuously in Badila, and were increased significantly at 16 h. These three factors maintained a small increase in ROC22 until 32 h, when the MDA content as well as POD and CAT enzyme activities were decreased in ROC22, indicating that it was less affected by stress in the later stage of drought (Figure 6A–C). The H_2_O_2_ content in Badila leaves was decreased continuously after 4–16 h of drought stress, increased again after 16–32 h. In ROC22, the H_2_O_2_ content of leaves was increased significantly after 4 h of drought stress, and then decreased and equable at the remain time points (Figure 6D).

“Peroxisome organization” and “glutamine family amino acid biosynthetic” processes were specifically up-regulated and enriched after sugarcane drought (Figure 3C). We analyzed genes related to antioxidants and found that the *FeSOD* gene was inhibited in the two genotypes during the later stage of stress treatment, but the *Cu*/*ZnSOD* gene was induced in RDT16, RDT32 and BDT32, indicating that different types of SOD enzymes participate in antioxidant defense in sugarcane materials under drought stress (Figure 6D). With increasing stress treatment time, the expression level of 9 *CAT* genes showed a continuous upward trend, and the expression level in ROC22 was higher than that in Badila, which showed that the CAT enzyme actively participated in the elimination of reactive oxygen free radicals in sugarcane exposed drought stress, and its performance in ROC22 was better than that in Badila (Figure 6D).

Plants have many genes encoding peroxidases (PRX), which participate in processes such as cell wall hardening, cross-linking of cell wall components, defending against pathogen infection and removing H_2_O_2_. Here we identified 35 categories of peroxidases (PRX) genes that included a total of 102 genes (Figure S1). The largest category was the *PRX1* gene, which had 55 members. Due to the large number of members identified, expression of the PRX genes in general was both up-regulated and down-regulated in sugarcane after stress, and the expression amount (FPKM) of up-regulated DEGs was generally higher than that of down-regulated DEGs. This finding shows that peroxidase also plays a role in scavenging ROS in sugarcane under drought stress (Figure 6E).

GPX is an essential part of antioxidant defense enzymes in plants. Under untreated conditions, a higher *GPX* expression pattern was seen for Badila relative to ROC22. However, with increasing drought stress treatment time, the *GPX4* (Sspon.01G0039510-2C) gene was up-regulated by more than 2-fold in RDT32, whereas little change in expression was seen for BDT32 (Figure 6E).

### 2.7. Sugarcane Carbohydrate Metabolism under Drought Stress

According to the previous analysis, we learned that the carbohydrate metabolism related processes (glycolytic process, sucrose biosynthetic process, glucose transport, monosaccharide transport and galactolipid biosynthetic process) were significantly enriched in multiple samples (Figure 3C and Table 1). Therefore, we made a detailed analysis of the genes related to the process.

**Figure 7:**
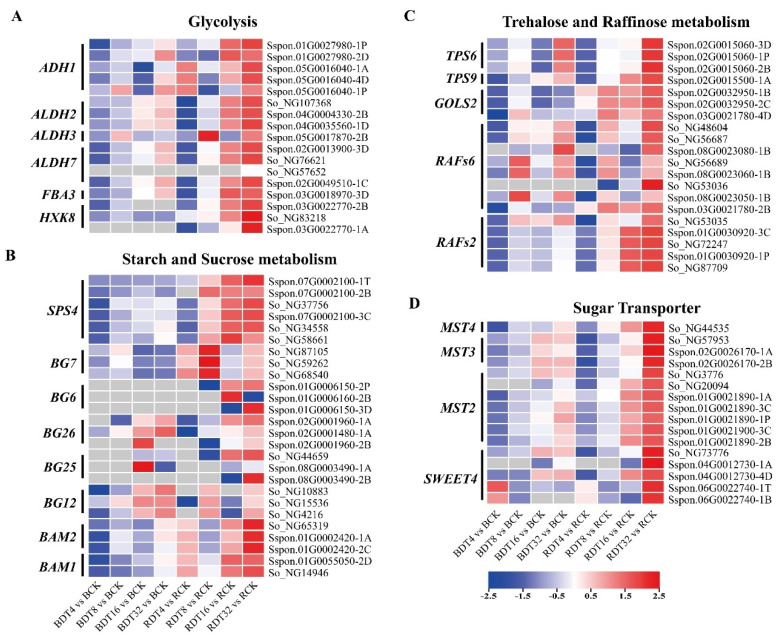
Carbohydrate metabolism related genes in sugarcane under drought stress. (**A**) Glycolysis. (**B**) Starch and Sucrose metabolism. (**C**) Trehalose and Raffinose metabolism. (**D**) Sugar Transpoter. The mapping data is by log_2_(FC), and the data were row standardized. Red is the up-regulated expression and blue is the down-regulated expression.

Glycolysis is an important carbohydrate metabolism pathway. The enzymes that catalyze this process act not only as catalysts and energy regulators, but also in signal transduction in response to environmental changes [15]. Analysis of the transcriptome data showed that the genes for key enzymes in glycolysis: *FBA3*, *ADH1*, *ALDH* and *HXK8*, which encode fructose diphosphate aldolase, alcohol dehydrogenase, aldehyde dehydrogenase and hexokinase, respectively, in sugarcane were up-regulated by drought stress, and that these genes had higher expression and up-regulation times in ROC22 compared with Badila (Figure 7A). Notably, *HXK8* was up-regulated only in ROC22 exposed to drought stress.

Sucrose-phosphate synthase (SPS) is the key enzyme of sucrose synthesis. We found that 12 *SPS4* genes were up-regulated in ROC22 exposed to drought stress, indicating that the sucrose content in ROC22 may increase under drought stress. β-Amylase (BAM) can break down instantaneous starch produced during the day and night to provide carbon and energy for plants. We found that under drought stress, multiple *BAM* genes in ROC22 had up-regulated expression, and the expression was always higher than that of Badila (Figure 7B). β-Glucosidase (BG) can hydrolyze intracellular ABA-glucose ester (ABA-GE) to produce ABA [16]. We found that several *BG* genes including *BG6*, *BG7*, *BG12, BG16*, *BG25* and *BG26* were differentially expressed genes in sugarcane under drought stress. Among these genes, levels of only two *BG12* were higher for ROC22 than Badila, and the remainder had lower levels in ROC22 than Badila (Figure 7B).

Trehalose 6-phosphate synthase (TPS) is a key enzyme in trehalose biosynthesis. We found that 16 *TPS* genes were up-regulated in sugarcane exposed to drought stress (Figure 7C and Figure S2, Table S4). Galactinol synthase (GOLS) and raffinose synthase (RAFs) are the key enzymes of raffinose synthesis [17]. *GOLS2* genes were up-regulated in sugarcane exposed to drought stress, and the expression amount and fold-up-regulation of ROC22 were higher than that of Badila. Seventeen *RAFs* genes were up-regulated in sugarcane induced by drought stress, and fourteen had dominant expression in ROC22, including *RAFs2* and *RAFs6* (Figure 7C and Figure S2, Table S4).

Sucrose transport mediates the distribution of photosynthetic products in plants in a key physiological process that is affected by drought and salt stress, because sucrose is the main energy and signal molecule and can penetrate membranes [18]. *SWEET4* encodes the sugar transporter in sugarcane and was up-regulated after drought stress; the expression amount and fold-up-regulation in ROC22 were higher than that in Badila. *MST* genes encoding monosaccharide transporters were also differentially expressed in sugarcane after drought stress, indicating that sugarcane may have an altered carbon distribution in order to adapt to drought stress, and the more severe differential expression of multiple of *MST* genes in ROC22 indicates that this adaptive response may be more prominent in this genotype (Figure 7D).

Comprehensive analysis showed that genes related to glycolysis, sucrose synthesis, starch degradation and raffinose synthesis were dominantly expressed or specifically expressed in ROC22. It shows that the participation of these pathways may be an underlying reason for the high drought resistance of ROC22.

### 2.8. Sugarcane Lipid Metabolism under Drought Stress

According to the previous analysis, we learned that the lipid metabolism related processes (membrane lipid biosynthetic process, glycophospholipid biosynthetic process and phospholipid biosynthetic process) were significantly enriched in multiple samples (Figure 3C). Therefore, we analyzed the genes related to lipid metabolism.

**Figure 8:**
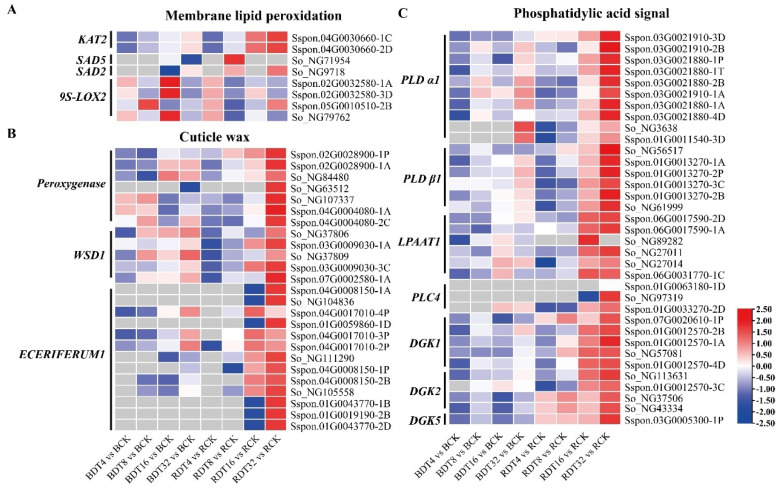
Lipid metabolism-related genes in sugarcane under drought stress. (**A**) Membrane lipid peroxidation. (**B**) Cuticle wax. (**C**) Phosphatidylic acid signal. The mapping data is by log_2_(FC), and the data were row standardized. Red is the up-regulated expression and blue is the down-regulated expression.

The accumulation of reactive oxygen species caused by drought stress will lead to membrane lipid peroxidation, reduce the content of unsaturated fatty acids (linoleic acid and α-linolenic acid), and then increase the cell membrane permeability. We found that 3-ketoacyl-CoA thiolase-2 (*KAT2*) [19] genes were up-regulated in sugarcane induced by drought stress, and had high expression and up-regulation multiple in ROC22 (Figure 8A). Stearoyl acyl carrier protein desaturase (SAD) is the key enzyme for the formation of unsaturated fatty acids [20]. We found that two *SAD* genes were up-regulated in sugarcane under drought stress. In addition, they were up-regulated in ROC22 at 8 h of stress, while Badila was up-regulated at 16 h of stress (Figure 8A). Linoleate 9S-lipoxygenase (9S-LOX) is an enzyme responsible for the oxidation of linoleic acid [21]. We found that four *9S-LOX* genes in sugarcane were inhibited and down-regulated under drought stress, and the expression of three genes in all samples of ROC22 was lower than that of Badila (Figure 8A).

Plant cuticle wax is composed of water insoluble lipid substances and covers the plant surface to control plant water transpiration and drought resistance. Peroxidase is a key enzyme involved in the formation of the cuticle matrix [22]. We found up-regulation of seven peroxidase genes in sugarcane exposed to drought stress. Of these, three were DEGs that were preferentially expressed in Badila under stress, and the other four had higher expression in ROC22 (Figure 8B). Wax ester synthase (WSD) is the key enzyme for cuticle wax synthesis [23]. We found that five *WSD1* genes were up-regulated in sugarcane in ROC22 exposed to drought stress, but only three were up-regulated in Badila. The up-regulation and expression was markedly higher in ROC22 compared to Badila (Figure 8B). *ECERIFERUM1* is the skeleton gene of wax biosynthesis [24]. We found 13 *ECERIFERUM1* genes that were up-regulated in sugarcane exposed to drought stress. Of these, 3 were DEGs that had preferential expression in Badila, whereas 10 had higher expression in ROC22 (Figure 8B).

Phospholipid acid (PA) is a lipid signaling molecule. As a second messenger, PA can accumulate rapidly in response to a variety of abiotic stress stimuli [25]. We found that a variety of genes related to PA synthesis were differentially expressed in sugarcane under drought stress (Figure 8C). Two Phospholipase D (*PLD*) genes, *PLD α* and *PLD β*, were induced up-regulated in sugarcane exposed to drought stress and this up-regulation was substantially higher in ROC22 compared to Badila; *PLD β* expression was significantly up-regulated only in ROC22 (Figure 8C). Lysophosphatidic acid acyltransferase (LPAAT) is a key enzyme in another PA synthesis pathway. Among genes encoding *LPAAT1*, six were up-regulated only in ROC22 exposed to drought stress (Figure 8C). Phospholipase C (PLC) and diacylglycerol kinase (DGK) are the key enzymes of a third PA synthesis pathway. We found up-regulation of 3 *PLC* genes in sugarcane under drought stress. Of these, two were up-regulated only in ROC22, and the expression amount and up-regulation of the three genes was higher for ROC22 than Badila (Figure 8C). *DGK* genes were up-regulated in sugarcane. The number of up-regulated *DGKs* of ROC22 were more than that of Badila, and the up-regulated expression began earlier (Figure 8C).

### 2.9. Regulatory Network of Sugarcane in Response to Drought Stress

The lavenderblush module contains 4323 DEGs, which was the most among all the modules. Functional enrichment analysis showed that genes in this module were significantly enriched in many processes related to abiotic stress response and signal transduction. The functional enrichment, expression pattern and construction of an expression network of genes in this module can help distinguish key drought regulators in sugarcane. Candidate transcription factors were screened according to Gene Significance (GS) > 0.2, Module Membership (MM) > 0.9 and module connectivity (kME) > 0.9. A total of 142 transcription factors were found, including 20 gene families (e.g., NAC, bZIP, MYB, AP2/ERF). The transcription factors with the top 150 kME values were selected as candidate key transcription factors (Table S5), and include *NAC87* (NAC, Sspon.01G0008840-1A), *JAMYB* (MYB, Sspon.02G0028760-2B), *bHLH84* (bHLH, So_NG102144), *NAC21/22* (NAC, Sspon.02G0031110-2C), *HOX24* (HB-HD-ZIP, Sspon.04G0036740-1D) and *MYB102* (MYB-related, Sspon.02G0000080-1P).

**Figure 9:**
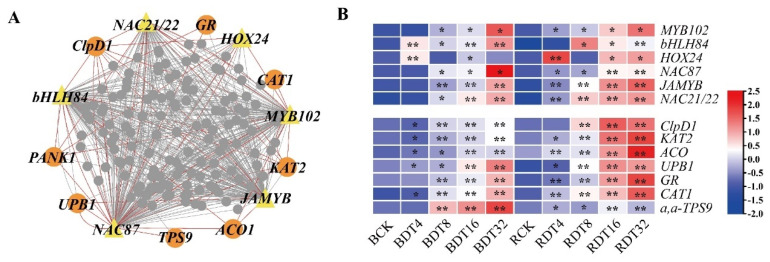
Co-expression network of transcription factors and structural genes related to drought stress response in sugarcane. (**A**) The co-expression network of transcription factors (yellow triangle) and structural genes (orange circle) related to the response process of drought stress, and the gray circle is the co-expression network of other genes related to transcription factors. (**B**) The heat map of drought stress related transcription factors and structural gene expression was drawn by qRT-PCR data. The data were standardized and logarithmic (the base number was 2). Red indicates high expression and blue indicates low expression. Student t test is used for significance test. All samples of Badila were compared with BCK, and all samples of ROC22 were compared with RCK, * indicates significant(*p* ≤ 0.05), ** indicates extremely significant(*p* ≤ 0.01).

Co-expression of *NAC87*, *JAMYB*, *bHLH84, NAC21/22*, *MYB102* and *HOX24* with genes related to drought stress response in sugarcane was analyzed (Figure 9A and Table S3). The *UBP1* gene encoding Beta-ureidopropionase, a key enzyme of β-alanine synthesis, had a strong co-expression relationship with *NAC87*, *JAMYB*, *NAC21/22*, *MYB102* and *HOX24*. These five transcription factors and *bHLH84* are co-expressed with the pantothenate kinase gene *PANK1*, which is a key enzyme in CoA biosynthesis. *UPB1* and *PANK1* genes were significantly enriched in the “Pantothenate and CoA biosynthesis” pathway. These six transcription factors were co-expressed with the gene encoding chaperone protein ClpD1. The constitutive overexpression of rice ClpD1 protein enhanced tolerance of transgenic *Arabidopsis* plants to salt and drought stress. *NAC87*, *JAMYB*, *bHLH84, NAC21/22*, *MYB102* and *HOX24* are also co-expressed with glutathione reductase and catalase isozyme 1 (*CAT1*), which are both important antioxidants in plants. *JAMYB*, *bHLH84*, *NAC21/22*, *HOX24* and *MYB102* are co-expressed with the α,α-trehalose phosphate synthase gene. These genes are also co-expressed with the 3-ketoacyl-CoA thiolase 2 gene (*KAT2*) (Figure 9A and Table S3). The result of qRT-PCR showed that the above 10 genes were up-regulated in sugarcane induced by drought stress (Figure 9B).

The five genes having the highest kME value were So_NG7572 (malate synthase, *MLS*), Sspon.01G0036410-1P (branched chain amino acid aminotransferase 3, *BCAT3*), Sspon.04G0017450-4D (OUT-like cysteine protease, *OTULCP*), Sspon.04G0030660-1C (3-ketoyl CoA thiolase 2, *PKT2*) and Sspon.01G0021890-3C (Sugar transport protein MST2, *MST2*) (Figure S3). KEGG enrichment analysis of the genes associated with these five genes showed that they were significantly enriched in pathways related to amino acid metabolism, peroxidase, coenzyme A biosynthesis, fatty acid metabolism, unsaturated fatty acid biosynthesis and metabolism, glucose metabolism and carbon metabolism (Table S6).

## 3. Discussion

In this study, we compared the shared gene pool of drought sensitive and drought tolerant genotypes under drought stress. Those identified genes may be an important goal for drought tolerant breeding. Under long-term drought stress, Badila decreased viability earlier than ROC22, which is consistent with the observed changes of photosynthetic parameters. The transcriptome data of the two sugarcane genotypes in multiple stress time gradients were analyzed. It was found that the transcripts of the two sugarcane genotypes were similar in the early stage of stress, but different in the later stage.

Here, drought stress led to leaf shrinkage and water loss, and RWC decreased with the increase in the amount of stress time. Plasma membrane intrinsic proteins (PIPs) are a type of aquaporin (AQP). AQPs play a key role in controlling the flow of water into and out of plant cells. PIP water channels promote transmembrane movement of water and small uncharged solutes [26]. *OsPIP1;3* was shown to enhance absorption of soil water by the plant to improve resistance to water deficits. *SIP1;1* and *PIP1;2* deletion mutants in *Arabidopsis* had impaired pollen hydration in the pistil [27]. We found that expression of five *SsPIP1;2* was up-regulated after 16 and 32 h of stress in ROC22, and the overall expression abundance in ROC22 at multiple time points was higher than that of Badila, indicating that *SsPIP1;2* could play an important role in regulating leaf water content under drought stress in sugarcane (Figure S4).

Hormone regulation is another response mechanism of plants to drought stress. Abscisic acid (ABA) helps plants survive in drought by regulating closure of guard cells [28]. We observed significant changes in ABA synthesis, metabolism and transport components in sugarcane transcriptome after stress, including NCED, AAO and ABC transporters, indicating that ABA plays an important role in sugarcane drought response. The up-regulated expression of *ACS* and *ACO* genes, which are key enzymes of ethylene biosynthesis in sugarcane exposed to drought stress, indicates that drought stress may induce ethylene production in sugarcane. Moreover, the high expression of these genes in ROC22 indicates that ethylene may play a role in conferring the high drought tolerance of this genotype. Ethylene is known to promote stomatal closure by promoting production of ROS mediated by NADPH oxidase in stomatal guard cells [29]. Here, the gene *RBOHF* encoding NADPH oxidase was induced by drought stress in sugarcane, especially in ROC22. This result indicates that there may be ethylene-mediated regulation of stomatal opening and closing in sugarcane exposed to drought stress. BR signal transduction in plants begins when BR is sensed by the BRI1 receptor kinase on the cell surface that then activates transcription factors *BZR1* and *BES1* to induce stress tolerance [30]. Moreover, the gene *DWARF4* encoding the rate limiting enzyme Cytochrome P450 90B2 of BR biosynthesis was up-regulated in ROC22 exposed to drought stress, suggesting that drought stress may induce BR accumulation in ROC22. Some studies found that the *BdBRI1* deletion mutant of *Brachypodium distachyon* has a DWARF phenotype that has enhanced drought tolerance [31]. We also found that expression of the BRI1 receptor kinase gene was inhibited in the late stage of ROC22 drought stress, which indicates that the high drought resistance of ROC22 may be related to the down-regulated expression of *BRI1* gene under stress. BZR1 transcription factor homolog gene expression was also induced in ROC22 after drought stress, which further indicated that BR-mediated signal transcription and drought stress response may occur in ROC22.

A typical MAPK cascade consists of MAPK (MPK), MAPK kinase (MAPKK, MAP2K, MKK or MEK) and MAPK kinase kinase (MAPKKK, MAP3K or MEKK). H_2_O_2_ activates a specific group of Arabidopsis MAPKKKs (ANP1, ANP2 and ANP3) and then starts the phosphorylation cascade, resulting in the activation of *MPK6* and *MPK3* [32], which is consistent with the gene changes in sugarcane. This indicates that the signal generated by MAPK cascade is successfully transmitted to the downstream gene. Previous studies have reported that MAPK cascade is involved in the biosynthesis and signal transduction of JA, SA and ET, *MKK9* and *MPK3_6* are involved in the signal transduction process of ethylene, and *AtMPK6* and *AtMKK3* negatively regulate *AtMYC2* expression and control jasmonic (JA) signal transduction [33]. It was found that *MKK3* and *MPK6* in sugarcane were induced under drought stress, indicating that MAPK cascade may be involved in the signal transduction of JA and ET in sugarcane. Under high salt and dehydration stress, Arabidopsis *MKK4* deletion mutant is more sensitive than wild-type. *AtMKK4* mediates osmotic stress response by regulating *AtMPK3* activity [34]. Analysis found that sugarcane *MKK4_5* and *MPK3* were up-regulated in RDT32, indicating that this MAPK signal cascade may also exist in sugarcane.

ROS are toxic by-products produced by plants under drought stress [35]. In the face of increased ROS in cells under stress, plants will eliminate ROS by increasing detoxification proteins [5]. We found that the activities of CAT and POD in ROC22 and Badila increased under drought stress, indicating that sugarcane could resist oxidative stress influence caused by drought stress by improving the activity of intracellular antioxidant enzymes. It could be found that the activities of CAT and POD enzymes in ROC22 were higher than these in Badila in the untreated state and after 4 and 8 h of stress. CAT1 protein can help to protect the plant against reactive oxidant related environmental stresses and responds to ABA and osmotic stress [36]. After analyzing the transcriptome data, we found that several *CAT1* genes were induced to express in sugarcane under drought stress, especially in ROC22, suggesting that *CAT1* gene may play an important role in response of ROC22 to drought stress. In the late phase, the content of H_2_O_2_ in ROC22 was equable, but in Badila, it was still increased. These results indicated that ROC22 may have more lasting antioxidant capacity. ROS also play a role in signal transduction mechanism in the response to stress [37]. H_2_O_2_ as a kind of ROS produced by RBOHF could regulate guard cell signaling and stomatal closure [38,39]. In ROC22, we found that *SsRBOHF* gene was up-regulated by drought stress and the H_2_O_2_ content increased significantly after 4 h of stress, suggesting that the signal transduction mechanism could also occurred in drought tolerant in sugarcane.

Fructose diphosphate aldolase, alcohol dehydrogenase, aldehyde dehydrogenase and hexokinase are the key enzymes that play a catalytic role in glycolysis. Many studies have shown that the expression of genes encoding these enzymes is induced by a variety of abiotic stresses (e.g., high temperature, drought, salt and low temperature), and plants expressing these genes to higher levels show higher photosynthetic capacity, antioxidant and ABA sensitivity that increases the abiotic stress resistance relative to wild type [40,41,42,43]. Our study found that the genes encoding *FBA3*, *ADH1*, *ALDH* and *HXK8* were up-regulated by drought stress in sugarcane, and showed dominant expression in ROC22. We speculated that glycolysis may play an important role in the drought response of sugarcane and could contribute to the high drought resistance of ROC22. Under various environmental stresses, plants often re-mobilize starch to provide energy and carbon when photosynthesis may be limited. The released sugars and other derived metabolites support the growth of plants under stress and act as osmotic protectants and compatible solutes to reduce the negative effects of stress [44]. Hong et al. found that the sweet potato β-amylase gene *IbBAM1.1* was overexpressed in *Arabidopsis*, which improved the drought and salt tolerance of plants that overexpressed this gene through the regulation of ROS homeostasis and osmotic balance. In our data, we also found that β-amylase genes (BAM) in ROC22 were up-regulated, indicating that starch degradation may occur in ROC22 under drought stress. In soybean leaves, drought stress induces increased sucrose content [45]. Sucrose is also an important osmotic regulator in plants and sucrose phosphate synthase is a key enzyme of sucrose synthesis. Our study found up-regulation of 12 sucrose phosphate synthase genes in ROC22 exposed to drought stress. Through the up-regulated expression of β-amylase gene and sucrose phosphate synthase gene in ROC22 under drought stress, we speculate that drought stress induces increased sucrose content in ROC22 leaves, and the sugar, energy and other metabolites that are produced may contribute to ROC22 drought tolerance. In addition to de novo synthesis, the ABA synthesis pathway in plants also includes hydrolysis of intracellular ABA-GE (ABA glucosyl ester) by β-glucosidase (BG). The one-step reaction catalyzed by β-glucosidase in which ABA-GE is hydrolyzed to ABA is an ideal and important way to promote rapid increases in ABA content that is necessary for plants to meet physiological demands [46]. Some studies have found that the rice β-glucosidase gene *Os3BGlu6* improves drought tolerance by regulating stomatal closure [47]. Several *BG* genes were found to be up-regulated in sugarcane exposed to drought stress, suggesting that the BG also promotes ABA-GE hydrolysis to form ABA in sugarcane exposed to drought stress. The dominant expression of these genes in ROC22 may also lead to earlier and more durable ABA-mediated drought response in ROC22. The expression patterns of *SWEET* and *MST* genes were altered in sugarcane exposed to drought stress, indicating that sugarcane may have altered carbon distribution to adapt and survive drought stress. The more severe differential expression of multiple of *SWEET* and *MST* genes in ROC22 indicates that this adaptive response may be more prominent in the drought-tolerant genotype.

Fatty acid β-oxidation not only provides energy and a carbon skeleton for plants, but also causes production of ROS, which is involved in ABA signaling. Tao Jiang et al. [48] found that KAT2, as a key enzyme of fatty acid β-oxidation, participates in ABA signal transduction by regulating ROS production in plant cells. Our study also found that *KAT2* genes in both ROC22 and Badila were up-regulated under drought stress, indicating that *KAT2* may be involved in ABA signal regulation in sugarcane. SAD enzyme promotes formation of unsaturated fatty acids. After drought stress, up-regulated expression of the sad gene in ROC22 occurs earlier than in Badila, indicating that unsaturated fatty acid production in ROC22 and the protective effect of antioxidants on membrane lipids may arise earlier than in Badila. Under drought stress, *9S-LOX* genes in ROC22 and Badila were inhibited, and their expression in ROC22 was always lower than in Badila, indicating that the degree of oxidation of linoleic acid in ROC22 may be lower than that in Badila. Analysis of key enzyme genes of the phospholipid acid synthesis pathway suggests that there may be additional phospholipid acid (PA) synthesis pathways in ROC22 compared to Badila after stress. PA may be involved in signal transduction of sugarcane under drought stress, and the PA signal transduction process in ROC22 is activated earlier and is more intense.

Transcription factors are key players in the regulatory network of plants in response to adverse environmental stress. Through WGCNA analysis, we obtained candidate transcription factors related to sugarcane drought tolerance. *SsNAC87* is a NAC transcription factor that has high similarity to *Arabidopsis AtNAC046*. Mahmood et al. [49] showed that *Arabidopsis* overexpressing *NAC046* accumulated 30% more leaf epidermal wax than wild-type plants and also promoted the accumulation of cork. Expression of this gene was also induced by damage to leaves. Therefore, we speculate that as drought stress progresses, sugarcane would continue to increase expression levels of *SsNAC87*, which may lead to the accumulation of wax and cork in leaves, especially in ROC22. Most MYB proteins in plants belong to the R2R3-MYB subfamily. Up to 65% of MYB genes expressed in rice seedlings are reported to be differentially regulated under drought stress [50]. MYB family transcription factors play an important role in the transcriptional regulation of plants in response to drought stress. *SsJAMYB* and *SsMYB102* are two MYB family transcription factors and *OsMYB102* is homologous to *SsMYB102*. Overexpression of *OsMYB102* in *Arabidopsis* reduces the tolerance of transgenic plants to salt stress and drought stress, but this gene is not overexpressed in rice. Piao et al. [51] found that *OsMYB102* delays leaf senescence by inhibiting ABA accumulation and signal transduction. The expression of *SsMYB102* in the two genotypes examined here continued to increase with increasing drought stress time, indicating that this gene may also play a regulatory role in the drought response of sugarcane. Overexpression of the *SsJAMYB* homolog *JAmyb* in transgenic *Arabidopsis* improved tolerance to high salt stress during seed germination, seedling growth and root elongation, and participated in the abiotic stress response mediated by JA [52]. *SsJAMYB* expression was up-regulated in sugarcane exposed drought stress, and the transcript abundance in ROC22 was much higher than in Badila, indicating an active involvement of *SsJAMYB* in regulating drought resistance in sugarcane.

## 4. Materials and Methods

### 4.1. Plant Materials and Library Preparation

The *Saccharum officinarum* L. Badila (weak drought resistance) and sugarcane cultivar ROC22 (strong drought resistance) from the National Sugarcane Engineering and Technology Research Center were selected in this study. Healthy, single bud sugarcane stems with a length of approximately 8 cm were cultivated as plants. Plants were placed in 10 cm diameter black, square basins containing nutrient soil. Sugarcane plants were grown in a growth chamber at 30 °C/28 °C with a 16 h/8 h light/dark cycle and 60% humidity. Water was provided once every 2 days until the leaves emerged and then was provided twice a day. Each pot received 50 mL of water. Drought stress treatment began when the plants grew to the 3–5 leaf stage. We selected sugarcane seedlings with uniform growth and began by removing the root soil. Plants were hydroponically cultured in water for 1 week and then 20% PEG6000 was added to simulate drought stress. The treatment gradients were 0, 4, 8, 16 and 32 h, and three biological experiments were assessed. Leaf samples were collected at each time point, frozen in liquid nitrogen and stored at −80 °C for RNA extraction.

### 4.2. RNA-Seq and Data Analyses

The second leaves from five independent plants were collected and pooled as one biological replicate for RNA-Seq at 0, 4, 8, 16 and 32 h after drought stress treatment. Three biological replicates were performed. Total RNA was isolated from plant leaves using Trizol reagent (Invitrogen, Burlington, ON, Canada). After the samples passed the quality test, the library was constructed. The first cDNA strand was synthesized with six base random primers (random hexamers) based on the mRNA template and then the second cDNA strand was synthesized by adding buffer, dNTPs, RNase H and DNA polymerase I. The cDNA was purified using AMPure XP beads. The purified, double-stranded cDNA was subjected to terminal repair. A tail and sequencing connector was added, and then the fragment size was selected with AMPure XP beads. Finally, the cDNA library was enriched by PCR. Sequencing was performed on a PE150 Illumina platform and the sequencing was completed by Biomarker Technologies Co., Ltd. (Beijing, China).

Clean data were obtained by filtering the raw data and then aligned with the sugarcane reference genome (*Saccharum spontaneum* AP85–441 genome, http://sugarcane.zhangjisenlab.cn/sgd/html/index.html, accessed on 22 June 2021) using HISAT2 [53]. The StringTie (version 1.3.1, Baltimore, MD, USA) program was used to assemble the reads for comparison and obtain mapped data [54]. The gene expression level was normalized using the Fragments per Kilobase of transcript per Million mapped reads (FPKM) method. All of the downstream analyses were based on high-quality clean data. The differential expression analysis between the treatment group and the control group was carried out using DESeq2 (version 1.6.3, Boston, MA, USA) [55]. The screening criteria of differentially expressed genes (DEGs) were that FDR was less than 0.05 and the absolute value of the difference multiple FC (fold change) was greater than or equal to 2. The DEGs annotated to the go database were enriched and analyzed by topGO (version 2.8, Saarbrücken, Germany), and the FDR was less than 0.05. KEGG enrichment analysis was carried out according to KOBAS (2.0), and the *p*-value correction value KS was less than 0.05.

### 4.3. Co-Expression Network Construction

Based on the expression correlation pattern between differentially expressed genes, the weighted gene co-expression network (WGCNA) was analyzed [56]. All DEGs were analyzed, Log_2_ (FPKM + 1) was used as the input value, and the soft threshold was set to 22 (power = 22) to make the network suitable for scale-free topology. The minimum number of genes in the module was 30 (minModuleSize = 30), and the merging threshold of similar modules was 0.25 (MEDissThres = 0.25). After the network was generated, the connection of transcription factors and their regulatory target genes were preliminarily predicted. The network was mapped using Cytoscape 3.3.0. (Seattle, WA, USA) [57].

### 4.4. Physiological Indicator Measurements

#### 4.4.1. Relative Water Content of Leaves

The leaves were weighed at the following three time points, including immediately after cutting, completely immersed in distilled water away from light for 12 h, and dried in the oven at 80 °C to a constant weight after distilled water, then Wf, Wt and Wd data were obtained successively. The relative water content (RWC) of the leaves was determined using the formula: RWC (%) = (Wf − Wd)/(Wt − Wd) × 100.

#### 4.4.2. Net Photosynthetic Rate and Stomatal Conductance

The net photosynthetic rate and stomatal conductance were measured for the primary leaves of the treatment and control groups using the LI-6800XT portable photosynthesis system (LI-COR; Lincoln, NE, USA). For assessment, 5 technical repetitions and 3 biological repetitions were taken at 0, 4, 8, 16 and 32 h after the initiation of treatment.

#### 4.4.3. POD and CAT Enzyme Activities and MDA and H_2_O_2_ Content

Physiological indexes, including POD and CAT activities as well as MDA and H_2_O_2_ content, were determined using a kit according to the manufacturer’s instructions (Solarbio company; Beijing, China). All physiological indexes included 5 biological repetitions.

### 4.5. RT-qPCR

The cDNA of materials at different time points was used as the template. Primer information is provided in Table S1. The Sugarcane Cullin (CUL) gene is used as the internal reference gene. The ABIPRISM7500 (Applied Biosystems, Waltham, MA, USA) instrument and fluorescence quantitative PCR kit (Biomarker Technologies Co., Ltd., Beijing, China) were used for real-time fluorescence quantitative PCR (qRT-PCR). The reaction procedure was conducted according to the kit and instrument instructions. A melting curve analysis was carried out after the reaction ended and the 2^−ΔΔCT^ algorithm was used to analyze the experimental results [58].

### 4.6. Statistical Analysis

A one-way analysis of variance (ANOVA) in combination with Duncan’s multiple range test with a significance of differences of *p* ≤ 0.05 were conducted by IBM SPSS statistics version 20 software. Means indicate at least three biological replicates. The heatmap, Venn chart, column chart, etc. are completed through BMKCloud platform (http://www.biocloud.net/, accessed on 1 July 2021, Bi-omarker Technologies Co., Ltd., Beijing, China), TBtools software [59], R language 4.0.2 and Excel.

## 5. Conclusions

Here we provide evidence of drought response in sugarcane that is characterized by changes in primary metabolism (carbohydrate metabolism and lipid metabolism) caused by changes in photosynthesis, improvement in antioxidant defense capacity, and changes in ABA, ET and BR-mediated hormone signaling. The data also reveal the differences between drought-resistant and drought-susceptible genotypes under drought stress. Possible mechanisms for drought tolerance were explored and candidate genes related to drought tolerance in sugarcane were explored by using a co-expression network. The transcription factors *SsNAC87*, *SsJAMYB*, *SsbHLH84*, *SsNAC21/22*, *SsHOX24* and *SsMYB102* each may play an important role in drought stress tolerance.

## Figures and Tables

**Table 1:** Expression patterns and functional annotation of genes in different modules.

Module Name	Number of Genes	GO-Enriched (FDR ≤ 0.05)	Expression Trends (Average)
antiquewhite4 cornflowerblue RCK	2809 36	phospholipid biosynthetic process (GO:0046474) membrane lipid biosynthetic process (GO:0046467) glycerophospholipid biosynthetic process (GO:0046474) hydrogen peroxide biosynthetic process (GO:0050665) glycolytic process (GO:0006096)	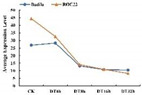
lightpink2 BCK	1390	phospholipid biosynthetic process (GO:0008654) membrane lipid biosynthetic process (GO:0046467) glycerophospholipid biosynthetic process (GO:0046474) galactolipid biosynthetic process (GO:0019375) galactolipid metabolic process (GO:0019374) regulation of cell growth (GO:0001558) cellular developmental process (GO:0048869)	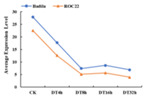
grey60 RDT4	1110	plant-type cell wall cellulose biosynthetic process (GO:0052324) cellulose biosynthetic process (GO:0030244) cellulose microfibril organization (GO:0010215)	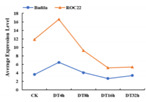
firebrick3 grey BDT4	67 52	glucose-6-phosphate dehydrogenase activity (GO:0004345)	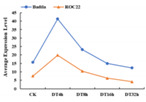
darkseagreen1 RDT8	157	regulation of brassinosteroid mediated signaling pathway (GO:1900457) negative regulation of brassinosteroid mediated signaling pathway (GO:1900458) steroid biosynthetic process (GO:0006694)	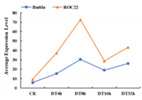
Brown1 BDT16	49	tRNA metabolic process (GO:0006399) intra-Golgi vesicle-mediated transport (GO:0006891) cis-Golgi network membrane (GO:0033106)	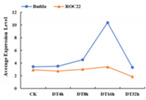
lavenderblush RDT32	4231	peroxisome (GO:0007031) glucose transport (GO:0015758) monosaccharide transport (GO:0015749) fatty acid beta-oxidation (GO:0006635) lipid oxidation (GO:0034440)	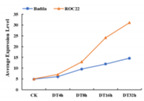

## Data Availability

Data is available at NCBI SRA accession PRJNA776107.

## Supplementary Materials

The following are available online at https://www.mdpi.com/article/10.3390/ijms23010569/s1.
