# Supplementary material for: Gene Co-Expression Analysis Reveals Transcriptome Divergence between Wild and Cultivated Sugarcane under Drought Stress
Source: Int J Mol Sci. 2022 Jan 5;23(1):569. doi: 10.3390/ijms23010569 (PMC8745624; doi:10.3390/ijms23010569)
Supplement: Supplementary file 1 [file ijms-23-00569-s001.zip › Supplementary Figure S1.pdf]

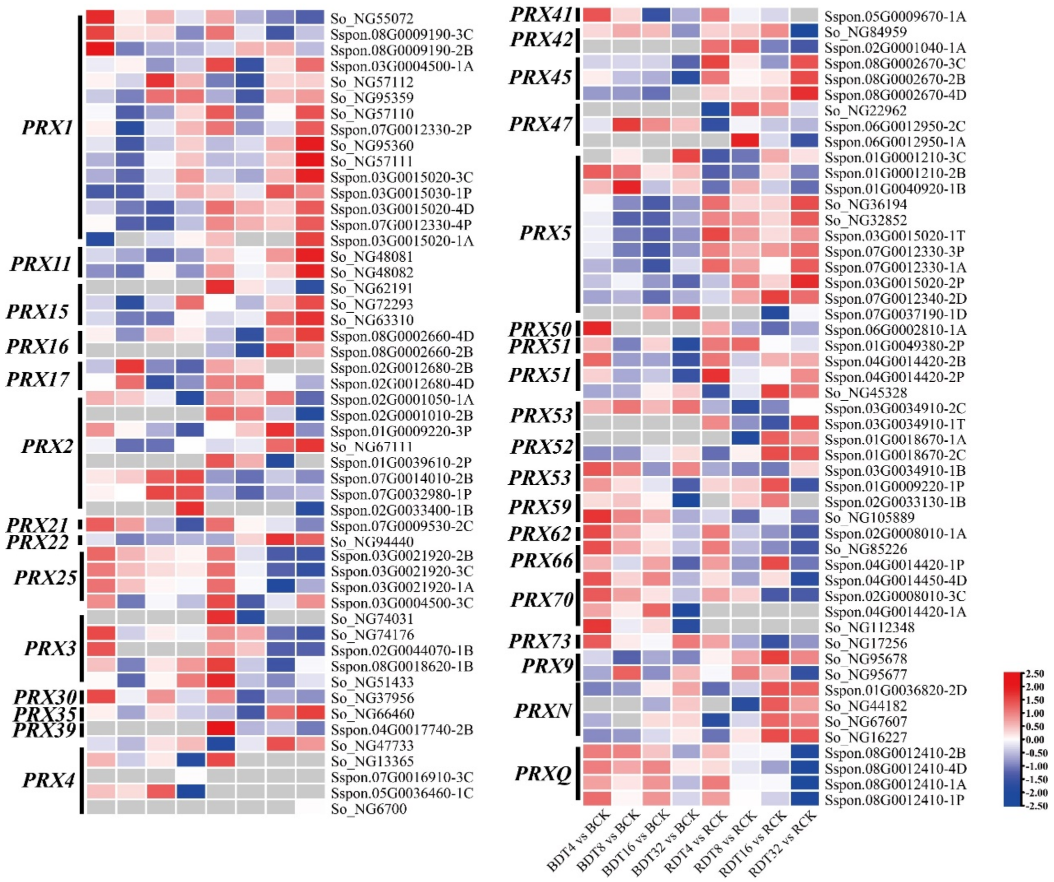

Supplementary Figure S1. Sugarcane PODs genes expression pattern. The heat map of genes was mapped with log_2(FC) and the data were row standardized. Red indicates up-regulated expression and blue indicates down-regulated expression.
