# Supplementary material for: Gene Co-Expression Analysis Reveals Transcriptome Divergence between Wild and Cultivated Sugarcane under Drought Stress
Source: Int J Mol Sci. 2022 Jan 5;23(1):569. doi: 10.3390/ijms23010569 (PMC8745624; doi:10.3390/ijms23010569)
Supplement: Supplementary file 1 [file ijms-23-00569-s001.zip › Supplementary Figure S3.pdf]

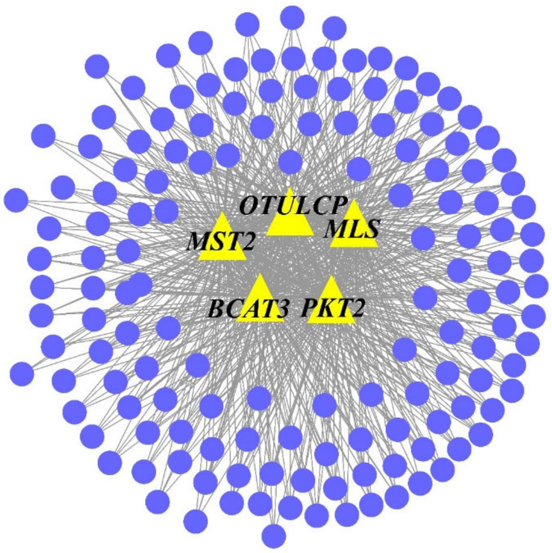

Supplementary Figure S3. Network diagram of DEGs with the first five KME values. The yellow triangle is the DEGs of the first five KME values, blue circles indicate genes that are related to candidate genes.
