# Supplementary material for: Gene Co-Expression Analysis Reveals Transcriptome Divergence between Wild and Cultivated Sugarcane under Drought Stress
Source: Int J Mol Sci. 2022 Jan 5;23(1):569. doi: 10.3390/ijms23010569 (PMC8745624; doi:10.3390/ijms23010569)
Supplement: Supplementary file 1 [file ijms-23-00569-s001.zip › Supplementary Table S1.pdf]

Supplementary Table S1. Primers for RT-qPCR

Gene ID	Gene Name	Forward sequence (5'-3')	Reverse sequence (5'-3')
Sspon.01G0008840-1A	*NAC87*	AGATGCAGTACCAGCAGCAG	CTTGCAGTACCTCCGGATCG
Sspon.02G0028760-2B	*JAMYB*	GCCGTCGTTTGATTATGATCAG	GGCCCAAAAGTTATCGGTAATG
So_NG102144	*bHLH84*	ACGCTGGTGATGATGAGCAA	ATCCTGTACAAAGATGGAGGTGT
Sspon.02G0031110-2C	*NAC21/22*	CAACATTGCCAGCAACAGCA	GATCAAAGCAGCCTCCCAGT
Sspon.04G0036740-1D	*HOX24*	GGTTGTCTTTGTGTATTCGAGG	GCATATACGTACACGCCAATAC
Sspon.02G0000080-1P	*MYB102*	CAACAACAACAACAACAACGTC	GTTGTTACATGTAGTCGCTCAC
Sspon.04G0030660-1C	*KAT2*	AGCTTTGCCGCTGTTGGAGT	AGCAGGAATGGCCACAGCAG
Sspon.02G0007510-2B	*UPB1*	TCTCGGCACCTGATGCCTCT	GCGGAAGGCCCACTTGTCTT
Sspon.08G0000830-1A	*CAT1*	CTAACAGGCTGCCGCGAGAA	TTGCCTGGCAGGGTCAAAGG
So_NG108312	*ClpD1*	CGCTCGGCCTTTGAGGAGAG	CGTGTCACCGGGCTTGAACT
Sspon.04G0021790-1P	*GR*	GCAGGCGTTGAGGTTGACCA	TGCTGGCCACCAAACACAGT
Sspon.02G0015500-4D	*TPS9*	CCCAGCTGACCTCCAACAGC	CCGAATTGACCGCCTCCACA
Sspon.03G0022400-1A	*ACO*	GACGGCAAAGTCTGCTGGGT	CCACCCGATGCTCCACACTC
	GAPDH	CACGGCCACTGGAAGCA	TCCTCAGGGTTCCTGATGCC
