# Supplementary material for: Gene Co-Expression Analysis Reveals Transcriptome Divergence between Wild and Cultivated Sugarcane under Drought Stress
Source: Int J Mol Sci. 2022 Jan 5;23(1):569. doi: 10.3390/ijms23010569 (PMC8745624; doi:10.3390/ijms23010569)
Supplement: Supplementary file 1 [file ijms-23-00569-s001.zip › Supplementary Table S2.pdf]

Supplementary Table S2. Overview of the RNA-seq data

Sample	Clean Reads (M)	Q30 (%)	Mapped Reads (M)(%)
BCK-1	30.49	93.30	45.95 (75.34)
BCK-2	36.42	93.43	55.36 (76.00)
BCK-3	31.79	93.15	46.60 (73.28)
BDT4-1	28.30	94.44	42.69 (75.43)
BDT4-2	29.44	94.58	46.28 (78.60)
BDT4-3	26.01	94.30	40.26 (77.38)
BDT8-1	27.72	94.37	39.37 (71.03)
BDT8-2	28.99	94.75	41.46 (71.51)
BDT8-3	30.32	94.44	43.91 (72.41)
BDT16-1	28.48	94.59	42.14 (73.98)
BDT16-2	28.85	94.11	44.43 (77.01)
BDT16-3	25.21	94.62	37.35 (74.06)
BDT32-1	37.85	93.64	59.96 (79.21)
BDT32-2	39.16	93.33	60.53 (77.29)
BDT32-3	41.94	93.72	63.05 (75.17)
RCK-1	27.51	94.38	46.52 (84.57)
RCK-2	36.45	94.38	63.05 (86.50)
RCK-3	29.35	94.14	49.90 (85.02)
RDT4-1	31.01	94.57	52.22 (84.20)
RDT4-2	30.78	94.44	51.45 (83.58)
RDT4-3	44.35	94.69	73.07 (82.38)
RDT8-1	33.47	94.43	55.17 (82.42)
RDT8-2	27.90	94.18	46.67 (83.64)
RDT8-3	28.20	94.58	46.75 (82.90)
RDT16-1	30.13	94.44	50.07 (83.10)
RDT16-2	33.27	94.54	54.15 (81.36)
RDT16-3	30.32	94.33	50.48 (83.25)
RDT32-1	30.26	94.29	51.28 (84.72)
RDT32-2	32.61	94.41	54.92 (84.20)
RDT32-3	35.70	94.34	60.30 (84.44)
