# Supplementary material for: Gene Co-Expression Analysis Reveals Transcriptome Divergence between Wild and Cultivated Sugarcane under Drought Stress
Source: Int J Mol Sci. 2022 Jan 5;23(1):569. doi: 10.3390/ijms23010569 (PMC8745624; doi:10.3390/ijms23010569)
Supplement: Supplementary file 1 [file ijms-23-00569-s001.zip › Supplementary Table S3.pdf]

Supplementary Table S3. GO Enrichment of RCK vs BCK differentially expressed gene

GO_ID	GO_Term	Items	Background Items	KS
	**Up DEGs**			
GO:0006952	defense response	55	1495	8.00E-28
GO:0007049	cell cycle	12	90	2.20E-25
GO:0034637	cellular carbohydrate biosynthetic process	16	246	2.10E-18
GO:0071555	cell wall organization	33	1138	4.10E-14
GO:0015979	photosynthesis	22	364	9.40E-13
GO:0009522	photosystem I	42	77	2.20E-15
GO:0009523	photosystem II	50	128	8.80E-14
GO:0000148	1,3-beta-D-glucan synthase complex	3	107	2.90E-11
GO:0016459	myosin complex	0	106	1.20E-08
GO:0009535	chloroplast thylakoid membrane	88	487	7.10E-08
GO:0043531	ADP binding	157	1559	< 1e-30
GO:0003678	DNA helicase activity	16	337	6.6E-27
GO:0005524	ATP binding	478	10712	9.1E-26
GO:0004803	transposase activity	9	80	1.7E-21
GO:0008270	zinc ion binding	149	3052	1.1E-17
	**Down DEGs**			
GO:0006313	transposition, DNA-mediated	10	90	1.6E-26
GO:0000723	telomere maintenance	12	246	2E-22
GO:0006310	DNA recombination	32	783	7.3E-19
GO:0015074	DNA integration	42	1138	6.4E-18
GO:0031047	gene silencing by RNA	13	452	1.1E-12
GO:0016459	myosin complex	5	106	5E-12
GO:0000148	1,3-beta-D-glucan synthase complex	1	107	8.5E-12
GO:0005663	DNA replication factor C complex	4	54	0.0000014
GO:0032153	cell division site	3	74	0.0000015
GO:0042555	MCM complex	0	73	0.0000022
GO:0005524	ATP binding	385	10712	< 1e-30
GO:0003678	DNA helicase activity	17	337	< 1e-30
GO:0043531	ADP binding	66	1559	2.4E-27
GO:0004803	transposase activity	10	80	7.1E-23
GO:0008270	zinc ion binding	98	3052	2.2E-19
