# Supplementary material for: Gene Co-Expression Analysis Reveals Transcriptome Divergence between Wild and Cultivated Sugarcane under Drought Stress
Source: Int J Mol Sci. 2022 Jan 5;23(1):569. doi: 10.3390/ijms23010569 (PMC8745624; doi:10.3390/ijms23010569)
Supplement: Supplementary file 1 [file ijms-23-00569-s001.zip › Supplementary Table S4.pdf]

Supplementary Table S4. Genes of trehalose 6-phosphate synthase (TPS), galactinol synthase (GOLS) and raffinose synthase (RAFs) in sugarcane under drought stress

ID	annotation	ID	annotation
Sspon.02G0015060-3D	Alpha,alpha-trehale-phphate synthase 6	Sspon.08G0023080-1B	Raffine synthase 6
Sspon.02G0015060-1P	Alpha,alpha-trehale-phphate synthase 6	Sspon.01G0030920-3C	Raffine synthase 2
Sspon.02G0015500-1A	Alpha,alpha-trehale-phphate synthase 9	So_NG72247	Raffine synthase 2
So_NG17861	Alpha,alpha-trehale-phphate synthase 9	So_NG53035	Raffine synthase 6
Sspon.02G0015060-2B	Alpha,alpha-trehale-phphate synthase 6	Sspon.01G0030920-1P	Raffine synthase 2
Sspon.02G0015500-2B	Alpha,alpha-trehale-phphate synthase 9	So_NG87709	Raffine synthase 2
Sspon.04G0001910-1P	Alpha,alpha-trehale-phphate synthase 11	So_NG56689	Raffine synthase 6
Sspon.04G0001910-4D	Alpha,alpha-trehale-phphate synthase 11	Sspon.08G0023060-1B	Raffine synthase 6
Sspon.04G0001910-3C	Alpha,alpha-trehale-phphate synthase 11	So_NG53036	Raffine synthase 6
So_NG59570	Alpha,alpha-trehale-phphate synthase 11	Sspon.08G0023050-1B	Raffine synthase 6
Sspon.02G0015500-4D	Alpha,alpha-trehale-phphate synthase 9	Sspon.03G0021780-2B	Raffine synthase 6
Sspon.08G0009350-1A	Alpha,alpha-trehale-phphate synthase 9	Sspon.01G0030920-2P	Raffine synthase 2
Sspon.02G0015500-2P	Alpha,alpha-trehale-phphate synthase 9	So_NG69321	Raffine synthase 2
So_NG21707	Alpha,alpha-trehale-phphate synthase 9	Sspon.01G0030920-2B	Raffine synthase 2
So_NG63096	Alpha,alpha-trehale-phphate synthase 9		
Sspon.02G0015500-1P	Alpha,alpha-trehale-phphate synthase 10		
Sspon.02G0032950-1B	Galactinol synthase 2		
Sspon.02G0032950-2C	Galactinol synthase 2		
Sspon.03G0021780-4D	Raffine synthase		
So_NG48604	Raffine synthase 6		
So_NG56687	Raffine synthase 6		
