# Supplementary material for: Gene Co-Expression Analysis Reveals Transcriptome Divergence between Wild and Cultivated Sugarcane under Drought Stress
Source: Int J Mol Sci. 2022 Jan 5;23(1):569. doi: 10.3390/ijms23010569 (PMC8745624; doi:10.3390/ijms23010569)
Supplement: Supplementary file 1 [file ijms-23-00569-s001.zip › Supplementary Table S5.pdf]

Supplementary Table S5. Candidate transcription factors related to drought stress response in sugarcane in lavenderblush module

GeneID	Family	kME	Module Membership	gene significance
Sspon.05G0028060-2D	AP2/ERF-ERF	0.97433	0.97433	0.83419
Sspon.05G0028060-1B	AP2/ERF-ERF	0.96133	0.96133	0.80079
Sspon.08G0012460-3C	AP2/ERF-ERF	0.93238	0.93238	0.77933
Sspon.08G0012460-1P	AP2/ER[1]F-ERF	0.94730	0.94730	0.76281
So_NG37349	AP2/ERF-ERF	0.96802	0.96802	0.74322
Sspon.05G0018650-2B	AP2/ERF-ERF	0.93652	0.93652	0.72551
So_NG40441	AP2/ERF-ERF	0.96678	0.96678	0.71913
So_NG110445	AP2/ERF-ERF	0.96374	0.96374	0.71823
So_NG43576	AP2/ERF-ERF	0.95880	0.95880	0.70457
Sspon.03G0002110-2D	AP2/ERF-ERF	0.96813	0.96813	0.70145
So_NG101395	AP2/ERF-ERF	0.96757	0.96757	0.67166
So_NG3786	AP2/ERF-ERF	0.94129	0.94129	0.61199
Sspon.07G0002770-1A	AP2/ERF-RAV	0.91266	0.91266	0.56688
So_NG85399	bHLH	0.95551	0.95551	0.83168
Sspon.05G0022410-2D	bHLH	0.91859	0.91859	0.82303
So_NG102144	bHLH	0.98947	0.98947	0.71762
So_NG60494	bHLH	0.93270	0.93270	0.67388
Sspon.07G0006610-1A	bHLH	0.94819	0.94819	0.66441
Sspon.04G0016220-3D	bZIP	0.93586	0.93586	0.87640
So_NG51751	bZIP	0.96897	0.96897	0.83084
So_NG45035	bZIP	0.92846	0.92846	0.79305
So_NG49903	bZIP	0.94987	0.94987	0.74309
Sspon.03G0009910-1A	bZIP	0.98237	0.98237	0.70836
Sspon.03G0009910-1P	bZIP	0.93359	0.93359	0.70489
Sspon.06G0007540-2C	bZIP	0.92502	0.92502	0.69211
So_NG37728	bZIP	0.96340	0.96340	0.67543
So_NG51149	bZIP	0.94080	0.94080	0.65283
Sspon.03G0009910-2P	bZIP	0.97459	0.97459	0.61528
Sspon.07G0001780-2P	bZIP	0.91869	0.91869	0.60629
So_NG104247	bZIP	0.92590	0.92590	0.60068
Sspon.06G0007540-1A	bZIP	0.96559	0.96559	0.59796
Sspon.03G0009910-3C	bZIP	0.93535	0.93535	0.58205
Sspon.03G0041470-1C	bZIP	0.90704	0.90704	0.58008
Sspon.06G0007750-1A	bZIP	0.96061	0.96061	0.57752
Sspon.07G0001780-1A	bZIP	0.90412	0.90412	0.55512
Sspon.06G0007540-3D	bZIP	0.91639	0.91639	0.42658
Sspon.03G0006610-2P	C2C2-GATA	0.90586	0.90586	0.59563
Sspon.02G0029820-2B	C2H2	0.95086	0.95086	0.84520
Sspon.02G0029820-4D	C2H2	0.94059	0.94059	0.81675
So_NG62848	C2H2	0.92215	0.92215	0.76878

So_NG9928	C2H2	0.97443	0.97443	0.74175
Sspon.01G0028770-3C	C2H2	0.91084	0.91084	0.68922
So_NG16846	C2H2	0.93203	0.93203	0.67442
Sspon.01G0057040-1C	C2H2	0.90590	0.90590	0.65084
So_NG2528	GARP-G2-like	0.91237	0.91237	0.90365
So_NG106483	GARP-G2-like	0.92094	0.92094	0.89338
Sspon.03G0025710-1B	GARP-G2-like	0.94926	0.94926	0.59885
Sspon.03G0025710-1P	GARP-G2-like	0.93200	0.93200	0.57738
So_NG96903	GARP-G2-like	0.92544	0.92544	0.52794
Sspon.04G0036740-1D	HB-HD-ZIP	0.98725	0.98725	0.73401
Sspon.04G0007240-2P	HB-HD-ZIP	0.93305	0.93305	0.72477
Sspon.04G0007240-3P	HB-HD-ZIP	0.93308	0.93308	0.70231
Sspon.04G0007240-1A	HB-HD-ZIP	0.91781	0.91781	0.66749
Sspon.04G0007240-2B	HB-HD-ZIP	0.90081	0.90081	0.60578
Sspon.02G0009520-3D	HB-HD-ZIP	0.92281	0.92281	0.56293
So_NG58070	HB-HD-ZIP	0.90935	0.90935	0.50274
Sspon.02G0004420-1P	HB-other	0.94742	0.94742	0.88387
Sspon.01G0005450-3D	HB-other	0.95214	0.95214	0.75486
Sspon.01G0005450-1A	HB-other	0.94843	0.94843	0.73654
Sspon.01G0005450-2B	HB-other	0.97267	0.97267	0.72010
So_NG79919	HB-WOX	0.92754	0.92754	0.62783
Sspon.01G0058310-1D	HB-WOX	0.92405	0.92405	0.53902
Sspon.02G0013260-1A	HSF	0.93896	0.93896	0.78084
Sspon.01G0037770-1B	LOB	0.96044	0.96044	0.85380
Sspon.02G0000080-2B	MYB	0.97762	0.97762	0.82025
Sspon.01G0038440-1P	MYB	0.92383	0.92383	0.81621
Sspon.03G0005140-2B	MYB	0.92950	0.92950	0.78089
Sspon.02G0028760-2B	MYB	0.99183	0.99183	0.76856
So_NG98580	MYB	0.91228	0.91228	0.75754
Sspon.03G0005140-1A	MYB	0.90520	0.90520	0.75512
Sspon.02G0028760-4D	MYB	0.96979	0.96979	0.71738
Sspon.06G0003930-4D	MYB	0.90491	0.90491	0.69771
So_NG85383	MYB	0.93066	0.93066	0.65164
Sspon.04G0016420-2B	MYB	0.91272	0.91272	0.64801
Sspon.02G0000760-3C	MYB	0.91289	0.91289	0.63887
Sspon.03G0019490-2B	MYB	0.90737	0.90737	0.61251
Sspon.02G0000760-4D	MYB	0.96971	0.96971	0.61145
Sspon.06G0031730-2D	MYB	0.96598	0.96598	0.60133
Sspon.03G0031760-2D	MYB	0.94499	0.94499	0.59968
So_NG20048	MYB	0.94150	0.94150	0.59574
Sspon.02G0028760-1T	MYB-related	0.97939	0.97939	0.83197
Sspon.02G0000080-1P	MYB-related	0.98365	0.98365	0.79466
Sspon.03G0036680-5P	MYB-related	0.91794	0.91794	0.78014
Sspon.02G0000080-1A	MYB-related	0.98266	0.98266	0.76970

Sspon.02G0028760-3C	MYB-related	0.93140	0.93140	0.73787
Sspon.02G0000080-3C	MYB-related	0.92389	0.92389	0.65794
So_NG84507	MYB-related	0.97191	0.97191	0.58811
Sspon.07G0006960-4D	MYB-related	0.93514	0.93514	0.55339
Sspon.07G0006960-2P	MYB-related	0.95605	0.95605	0.54040
Sspon.05G0020650-3C	NAC	0.90985	0.90985	0.93390
Sspon.05G0020650-1A	NAC	0.92260	0.92260	0.90059
Sspon.04G0009710-1P	NAC	0.94579	0.94579	0.88787
So_NG95774	NAC	0.95975	0.95975	0.88343
Sspon.04G0009710-3P	NAC	0.94781	0.94781	0.88342
Sspon.04G0009710-4P	NAC	0.92711	0.92711	0.87544
Sspon.03G0018230-1A	NAC	0.96352	0.96352	0.87377
Sspon.03G0018230-3D	NAC	0.96473	0.96473	0.86579
Sspon.01G0008840-3P	NAC	0.97367	0.97367	0.81044
Sspon.01G0008840-2B	NAC	0.94730	0.94730	0.80849
Sspon.01G0008870-1A	NAC	0.95522	0.95522	0.79645
Sspon.01G0008870-2B	NAC	0.95467	0.95467	0.79491
Sspon.01G0008870-4D	NAC	0.92521	0.92521	0.76741
Sspon.05G0020650-2P	NAC	0.94762	0.94762	0.76697
Sspon.03G0001860-1P	NAC	0.97458	0.97458	0.74723
Sspon.02G0031110-2C	NAC	0.98751	0.98751	0.74629
Sspon.01G0008840-1A	NAC	0.99502	0.99502	0.74347
So_NG33797	NAC	0.97755	0.97755	0.74190
Sspon.01G0008840-4P	NAC	0.97829	0.97829	0.73842
Sspon.01G0008840-2P	NAC	0.97857	0.97857	0.73465
Sspon.05G0020650-1P	NAC	0.94984	0.94984	0.73439
Sspon.02G0031110-1A	NAC	0.97122	0.97122	0.71624
Sspon.05G0020650-3P	NAC	0.98054	0.98054	0.70493
So_NG62385	NAC	0.97691	0.97691	0.69608
Sspon.01G0008840-1P	NAC	0.97877	0.97877	0.67761
So_NG84131	NAC	0.97666	0.97666	0.67726
Sspon.02G0027090-3C	NAC	0.90434	0.90434	0.66745
So_NG33798	NAC	0.92378	0.92378	0.63077
Sspon.02G0022250-4D	NAC	0.91943	0.91943	0.62185
Sspon.03G0001860-2B	NAC	0.95012	0.95012	0.59056
Sspon.01G0042440-1P	NAC	0.96576	0.96576	0.58478
Sspon.03G0001860-3C	NAC	0.95684	0.95684	0.58315
Sspon.07G0025430-1B	NAC	0.92045	0.92045	0.57936
Sspon.01G0042440-1B	NAC	0.96249	0.96249	0.57607
So_NG58969	NAC	0.90054	0.90054	0.43806
Sspon.03G0006000-1A	OFP	0.96351	0.96351	0.75817
So_NG92253	OFP	0.97435	0.97435	0.75461
Sspon.01G0004400-1A	Tify	0.90048	0.90048	0.92519
Sspon.07G0014340-3C	Trihelix	0.92091	0.92091	0.92494

Sspon.04G0007250-3C	Trihelix	0.93352	0.93352	0.88514
Sspon.05G0008580-2B	Trihelix	0.94935	0.94935	0.85608
Sspon.05G0008580-3C	Trihelix	0.96260	0.96260	0.82542
So_NG60866	Trihelix	0.95988	0.95988	0.81546
Sspon.03G0025350-4D	WRKY	0.95193	0.95193	0.75846
Sspon.06G0025040-1B	WRKY	0.93880	0.93880	0.68588
So_NG103504	WRKY	0.97690	0.97690	0.67998
Sspon.06G0001010-1A	WRKY	0.95145	0.95145	0.61405
Sspon.03G0036630-2C	WRKY	0.95375	0.95375	0.60698
So_NG61045	WRKY	0.93043	0.93043	0.59154
Sspon.06G0001010-3C	WRKY	0.91821	0.91821	0.57673
Sspon.06G0001010-2B	WRKY	0.92184	0.92184	0.57174
Sspon.01G0008580-2B	WRKY	0.93125	0.93125	0.55979
So_NG6401	zf-HD	0.95329	0.95329	0.84739
