# Supplementary material for: Gene Co-Expression Analysis Reveals Transcriptome Divergence between Wild and Cultivated Sugarcane under Drought Stress
Source: Int J Mol Sci. 2022 Jan 5;23(1):569. doi: 10.3390/ijms23010569 (PMC8745624; doi:10.3390/ijms23010569)
Supplement: Supplementary file 1 [file ijms-23-00569-s001.zip › Supplementary Table S6.pdf]

Supplementary Table S6. KEGG enrichment of genes with the first 150 kME values

Kegg_pathway	ko_id	Items	Gene_ID
Valine, leucine and isoleucine degradation	ko00280	13	So_NG107368;So_NG37445;So_NG56107;So_NG66496;So_NG66936;So_NG95465;Sspon.01G0036410-1P;Sspon.01G0036410-2C;Sspon.01G0057720-1D;Sspon.03G0015310-2B;Sspon.03G0018740-2C;Sspon.04G0030660-1C;Sspon.04G0030660-2D
Pantothenate and CoA biosynthesis	ko00770	8	So_NG107368;So_NG48422;So_NG66496;Sspon.01G0036410-1P;Sspon.01G0036410-2C;Sspon.01G0057720-1D;Sspon.02G0007510-2B;Sspon.02G0007510-3D
Propanoate metabolism	ko00640	7	So_NG37445;So_NG56107;So_NG66936;So_NG95465;So_NG97800;Sspon.03G0015310-2B;Sspon.03G0018740-2C
Peroxisome	ko04146	9	So_NG32819;So_NG5821;So_NG97800;Sspon.01G0007100-2C;Sspon.04G0030660-1C;Sspon.04G0030660-2D;Sspon.05G0019710-1P;Sspon.07G0008620-4D;Sspon.08G0000830-1A
Fatty acid degradation	ko00071	7	So_NG107368;So_NG60598;So_NG97800;Sspon.03G0018740-2C;Sspon.04G0030660-1C;Sspon.04G0030660-2D;Sspon.05G0019710-1P
Caffeine metabolism	ko00232	3	So_NG101430;So_NG5821;Sspon.03G0039740-1C
Glucosinolate biosynthesis	ko00966	4	So_NG66496;Sspon.01G0036410-1P;Sspon.01G0036410-2C;Sspon.01G0057720-1D
Fatty acid metabolism	ko01212	6	So_NG60598;So_NG97800;Sspon.03G0018740-2C;Sspon.04G0030660-1C;Sspon.04G0030660-2D;Sspon.05G0019710-1P
beta-Alanine metabolism	ko00410	5	So_NG107368;So_NG66936;So_NG97800;Sspon.02G0007510-2B;Sspon.02G0007510-3D
Valine, leucine and isoleucine biosynthesis	ko00290	4	So_NG66496;Sspon.01G0036410-1P;Sspon.01G0036410-2C;Sspon.01G0057720-1D
Glyoxylate and dicarboxylate metabolism	ko00630	6	So_NG7572;Sspon.01G0002150-1P;Sspon.03G0018740-2C;Sspon.05G0008290-2B;Sspon.05G0008290-4D;Sspon.08G0000830-1A
Lysine degradation	ko00310	5	So_NG107368;So_NG108685;Sspon.03G0018740-2C;Sspon.04G0002070-1A;Sspon.04G0002100-1A
Carbon metabolism	ko01200	9	So_NG66936;So_NG7572;So_NG97800;Sspon.01G0002150-1P;Sspon.03G0018740-2C;Sspon.05G0006050-2B;Sspon.05G0008290-2B;Sspon.05G0008290-4D;Sspon.08G0000830-1A
2-Oxocarboxylic acid metabolism	ko01210	5	So_NG66496;Sspon.01G0002150-1P;Sspon.01G0036410-1P;Sspon.01G0036410-2C;Sspon.01G0057720-1D
Biosynthesis of unsaturated fatty acids	ko01040	3	So_NG97800;Sspon.04G0030660-1C;Sspon.04G0030660-2D
Pyruvate metabolism	ko00620	5	So_NG107368;So_NG7572;Sspon.03G0018740-2C;Sspon.05G0008290-2B;Sspon.05G0008290-4D
